# Introducing DermExpoDB: a collaborative online database software application for occupational dermal exposure data

**DOI:** 10.3389/fpubh.2026.1765605

**Published:** 2026-06-03

**Authors:** Janine Schwarz, Urs Schlüter, Dag Rother, Daniel Vetter, Marcel Schumann, Suzanne Spaan, Ilse Ottenbros, Remy Franken, Rianda Gerritsen-Ebben, Violaine Verougstraete, Jessica Meyer

**Affiliations:** 1 BAuA, Dortmund, Germany; 2 EBRC Consulting GmbH, Hannover, Germany; 3 TNO, Utrecht, Netherlands; 4 European Metals, Brussels, Belgium

**Keywords:** dermal exposure data, dermal exposure database, dermal monitoring, dermal occupational exposure, dermal workplace exposure, FAIR principle, harmonized data format

## Abstract

DermExpoDB (https://dermexpodb.baua.de/) is a new web-based database software application developed through a collaboration between the German Federal Institute for Occupational Safety and Health (BAuA), the Netherlands Organisation for Applied Scientific Research (TNO), EBRC Consulting GmbH (EBRC), and European Metals (Europe’s non-ferrous metals association). It aims to collect, organize, harmonize, and share occupational dermal exposure data (implementing the FAIR principle for research data sharing as far as possible) to support exposure research, model development and evaluation, and workplace risk assessment for chemicals. DermExpoDB offers extensive contextual data fields, preserves data ownership, and provides tiered access to researchers and the public. This paper introduces the structure and functionality of DermExpoDB, along with the consortium’s vision to advance dermal exposure science and occupational health protection.

## Introduction

Dermal exposure to hazardous substances is a key but often underrepresented component in workplace risk assessments due to limited availability and accessibility of relevant data. While numerous databases exist for toxicological, environmental and workplace air exposure data (1), dermal exposure measurements remain dispersed, which limits their use in exposure and risk assessments for workplaces. The organized collection and storage of data on skin exposure currently takes place internally by only a few institutes, with limited exchange between them and other stakeholders. Database initiatives from the 2000s have not been updated, are technically outdated and are no longer publicly accessible; e.g. the Bayesian Exposure Assessment Toolkit (BEAT) of the British Health & Safety Executive (HSE) (2) or RISKOFDERM (3).

To address this gap, the German Federal Institute for Occupational Safety and Health (BAuA), in collaboration with the Netherlands Organisation for Applied Scientific Research (TNO) and EBRC Consulting GmbH (EBRC), and European Metals, has developed DermExpoDB (Dermal Exposure Database), a web-based database software application designed to collect, manage, and facilitate the sharing of occupational dermal exposure data. This paper introduces the features and use cases of DermExpoDB, and highlights its role in supporting dermal exposure research and model development.

DermExpoDB serves four primary purposes:

1. To define a format for collection and organisation of dermal exposure data consisting of the dermal exposure measurements, contextual information such as sampling strategy and method, study design, work activity, and other relevant information,
2. To enable the exchange of occupational dermal exposure data between cooperating institutes for exposure research and exposure model development/evaluation,
3. To provide external researchers and stakeholders access to occupational dermal data for scientific purposes, and
4. To present selected information on occupational dermal exposure, such as summary statistics, to the public domain.

## Methods

The development of DermExpoDB was carried out in three phases.

Phase 1 focused on organizing the available information on dermal data into dedicated data fields. A preliminary data structure was developed, based on a codebook that was initially developed by TNO and further advanced by all partners. This involved defining variable names, descriptions, and data field types.

In Phase 2, the DermExpoDB codebook was tested and refined using selected real-world data collected by BAuA. A graphic representation of the database structure can be found in the Supplementary materials 2, 3, and 5. Value labels were defined, and obligatory and optional fields were introduced to address missing information. (Complete lists of data fields for raw and summary data can be found in Supplementary materials 4, 6). Datasets meeting all obligatory requirements can be included, while missing optional fields is accepted. Additional descriptive text and help content for the database were also created (Supplementary material 7).

In addition, principles for sharing data were established within DermExpoDB, resulting in distinct software access levels and procedures for requesting data and metadata access. Also, two separate agreement documents in the collaboration were developed in Phase 2: the ‘Cooperation Agreement’ for data owners wishing to upload dermal datasets to DermExpoDB, and the ‘User Agreement’ which defines the terms for data sharing between data users and data owners.

Phase 3 involved developing and testing the database software application in Drupal, a free and open-source web content management system (https://www.drupal.org/about), with the programming carried out by a contracted external service provider. The programming phase was carried out with close coordination and input from the project partners. Applicable dermal exposure data and metadata were converted into the DermExpoDB format, and uploaded by the project partners.

### Results—key features of the database

Key features of DermExpoDB are described below. These key features include, amongst others, data content types and data ownership, with the aim of implementing the FAIR principles for research data, namely findability, accessibility, interoperability and reusability (4). It should be noted that FAIR data is not the same as open data. Regarding the data in DermExpoDB, ownership and intellectual property rights remain with the respective data owners. A FAIR self-assessment of the database by the Australian Research Data Commons (ARDC) resulted in a score of 59% (Supplementary material 10).

*Data content types*: Two types of workplace dermal exposure data can be uploaded in DermExpoDB: *raw datasets* and *summary statistic datasets*. Raw datasets consist of exposure measurements for a specific worker, volunteer or individual at a specific point in time and location. These raw datasets are usually the result of dermal monitoring studies carried out by or in collaboration with the data owner. Summary statistics datasets consist of statistical values reported in the original studies. These values are derived from underlying measurement datasets that typically comprise exposures for several workers, volunteers or individual persons within a defined exposure situation.

*Data ownership*: Authorship of all content, files, texts and graphics in DermExpoDB varies on a case-by-case basis and is governed by generally accepted conventions for scientific publications. The respective data owners of raw datasets expressly reserve all publication, reproduction, processing and exploitation rights to the content. Specifically, data owners maintain the proprietary rights over their datasets and metadata stored in DermExpoDB. While data owners are generally willing to share the dermal datasets stored in DermExpoDB, they reserve the right to withhold certain datasets or reject specific data requests. This enables data owners to retain control over their datasets and metadata if necessary to prevent potential misuse.

Each dataset in DermExpoDB includes not only the dermal measurement results for different body parts but also extensive metadata categorized into two types:

- Intrinsic metadata include information such as content type, data owner, upload status and upload / modification date.
- Contextual metadata comprise information related to the dataset itself and are logically organized in various sections within the database scheme. The sections organize information relating to references, activities performed, workplace conditions, implemented control measures, substances, formulations and sampling strategies, as well as measurement-specific details.

*Findability*: Each dataset in DermExpoDB is assigned a unique identifier within the system to ensure unambiguous referencing and internal data management. DermExpoDB also provides an advanced search functionality that allows users to identify groups of sub-datasets that fulfil the specified search properties (e.g., measurements of specific substances or with specific sampling methods) by querying up to 21 predefined data fields. Data owners must provide information for all these 21 data fields, and additional 9 fields, when uploading their datasets (see Table 1 for a complete list of the mandatory data fields and search fields in DermExpoDB). The fields for summary data and raw data are largely identical, with the exception of the fields for entering measurement values.

**Table 1:** Obligatory data fields and search fields in DermExpoDB.

Category	Data field	Search field
Data owner	Data owner	✓
	Confidentiality	×
Dataset	Type of data (raw data or summary data)	✓
	Dataset title	×
	Date of entry	×
	Sampling strategy (e.g., shift-based)	✓
	Exposure route(s)	✓
Study	Reference title	✓
	Digital identifier (e.g., DOI)	×
	Author(s)	✓
	Year of publication	✓
	Study quality	✓
Sampling	Study design (e.g. field study)	✓
	Sampling method potential hand exposure	✓
	Sampling method actual hand exposure	✓
	Sampling method potential body exposure	✓
	Sampling method actual body exposure	✓
Activity	Activity	✓
	Activity description—short	×
	Activity description—details	×
	PROC number & PROC description	✓
	Location	✓
Workplace	Workplace description	×
Control measures	Personal protective equipment	×
Product/substance/properties	Chemical substance/agent	×
	Vapour pressure	✓
	CAS	✓
	Product category	✓
	Product type	✓
	Chemical formulation/application solution/mixture type	✓

*Accessibility*: The database software application is accessible via a web-based frontend on the DermExpoDB website (https://dermexpodb.baua.de/). Individual users must create a personalised user account to access the interface. The database software application incorporates an extensive and detailed data structure based on a codebook developed by TNO. The underlying DermExpoDB codebook will be made available to data owners who wish to include their data in the database. The ability to map extensive contextual information enables highly detailed and differentiated analyses. For example, this can be used for customised development and calibration of dermal exposure models, or for the evaluation of existing dermal exposure models.

When uploading a dataset, data owners must complete a minimum of 30 obligatory fields for each data set, balancing detail with practicality.

*Interoperability*: All information in DermExpoDB is published in English. Each data field is assigned a unique machine-readable name. To facilitate external processing and analysis, many of the data fields require the selection from predefined categories. These categories are based on definitions from recognised external sources, such as the ART model (5), e.g., the definition of technical emission control at source or dispersion control away from source, and publications by ECHA, e.g., process categories (PROCs) and product categories (PCs) (6). For some data fields, the selectable categories were developed based on the data available at that time, as no other sources covered these fields. This applies to fields relating to personal protective equipment, hygiene measures, product types and sampling techniques. The vocabulary used for the data and metadata is widely recognised in exposure science, and any abbreviations or potentially ambiguous definitions are explicitly explained within DermExpoDB (Supplementary materials 8, 9).

*Reusability*: In most cases, the data in DermExpoDB has previously been published in the form of reports or scientific journal articles. Therefore, when uploading datasets, it is mandatory for data owners to clearly specify their origin. DermExpoDB offers several fields in the “Reference” section to facilitate this process.

These data fields were designed to enable all data owners or cooperation partners to translate their data into DermExpoDB with as little effort as possible. To facilitate future (semi-automated) external analysis and processing of dermal data, DermExpoDB includes several categorical fields. Additionally, DermExpoDB provides multiple free-text fields that allow data owners to provide detailed contextual information about the conditions under which the dermal datasets were generated, as well as outlining any study-related or dataset-specific limitations.

The usage rights of dermal exposure datasets and the corresponding metadata in DermExpoDB must be negotiated between the data owner and the data user during a data access request process, which follows a structured and regulated procedure. It is initiated by the data user through a request form within DermExpoDB and involves verification steps carried out by the data owner. The scope of these usage rights is formalized in a signed “Grant of Use Agreement”, which defines the rights of the data user to use the datasets. Use of datasets is generally restricted to the scientific purpose specified by the data users in their access requests and/or the usage rights negotiated between the data owner and the data user during the access request process.

Selected data analyses will be made publicly accessible to promote transparency and to disseminate knowledge on occupational dermal exposure stored within DermExpoDB.

*Data import and export*: After registration has been approved and the ‘Cooperation Agreement’ document is signed, users can easily import new datasets using a form or an interface that supports CSV file uploads.

Once a request to use a dataset has been approved, it can be exported for further analysis.

### Data base contents of DermExpoDB

As of February 2026, DermExpoDB contained 629 original raw datasets from BAuA and TNO projects. These are described in Table 2 and in the text below for the 619 BAuA data.

**Table 2:** BAuA projects in DermExpoDB.

BAuA project	Year	Project report	Publication(s)
*Arbeitsplatzbelastungen bei der Verwendung von Biozid-Produkten. Teil 1: Inhalative und dermale Expositionsdaten für das Versprühen von flüssigen Biozid-Produkten* Only available in German, translation: Occupational exposure when using biocidal products. Part 1: Inhalation and dermal exposure data for the spraying of liquid biocidal products	2004	https://www.baua.de/DE/Angebote/Publikationen/Berichte/Gd35.pdf	(7, 28)
*Arbeitsplatzbelastungen bei der Verwendung von bioziden Produkten. Teil 4: Holzschutzmittel* Only available in German, translation: Occupational exposure when using biocidal products. Part 4: Wood Preservatives	2009	https://www.baua.de/DE/Angebote/Publikationen/Berichte/F1809.pdf	(12)
*Validation of an EDP-assisted model for assessing inhalation exposure and dermal exposure during spraying processes*	2012	https://www.baua.de/DE/Angebote/Publikationen/Berichte/F2137.pdf	(8)
*Messung von Hautbelastungen durch chemische Stoffe bei der Imprägnierung mit Holzschutzmitteln* Only available in German, translation: Measurement of dermal exposure to chemical substances during impregnation with wood preservatives	2012	https://www.baua.de/DE/Angebote/Publikationen/Berichte/F2053	(13)
*Comparative study on exposure of workers and bystanders during pest control of the Oak Processionary Moth by spray application*	2017	https://doi.org/10.21934/baua:bericht20170718	(29)
*SysDEA: Systematic analysis of dermal exposure to hazardous chemical agents at the workplace*	2019	https://doi.org/10.21934/baua:bericht20190116	(10)
*Human exposure to biocidal products: Measurement of inhalation and dermal exposure during the application of biocide foams*	2022	https://doi.org/10.21934/baua:report20220106	(11)

**Figure 1:**
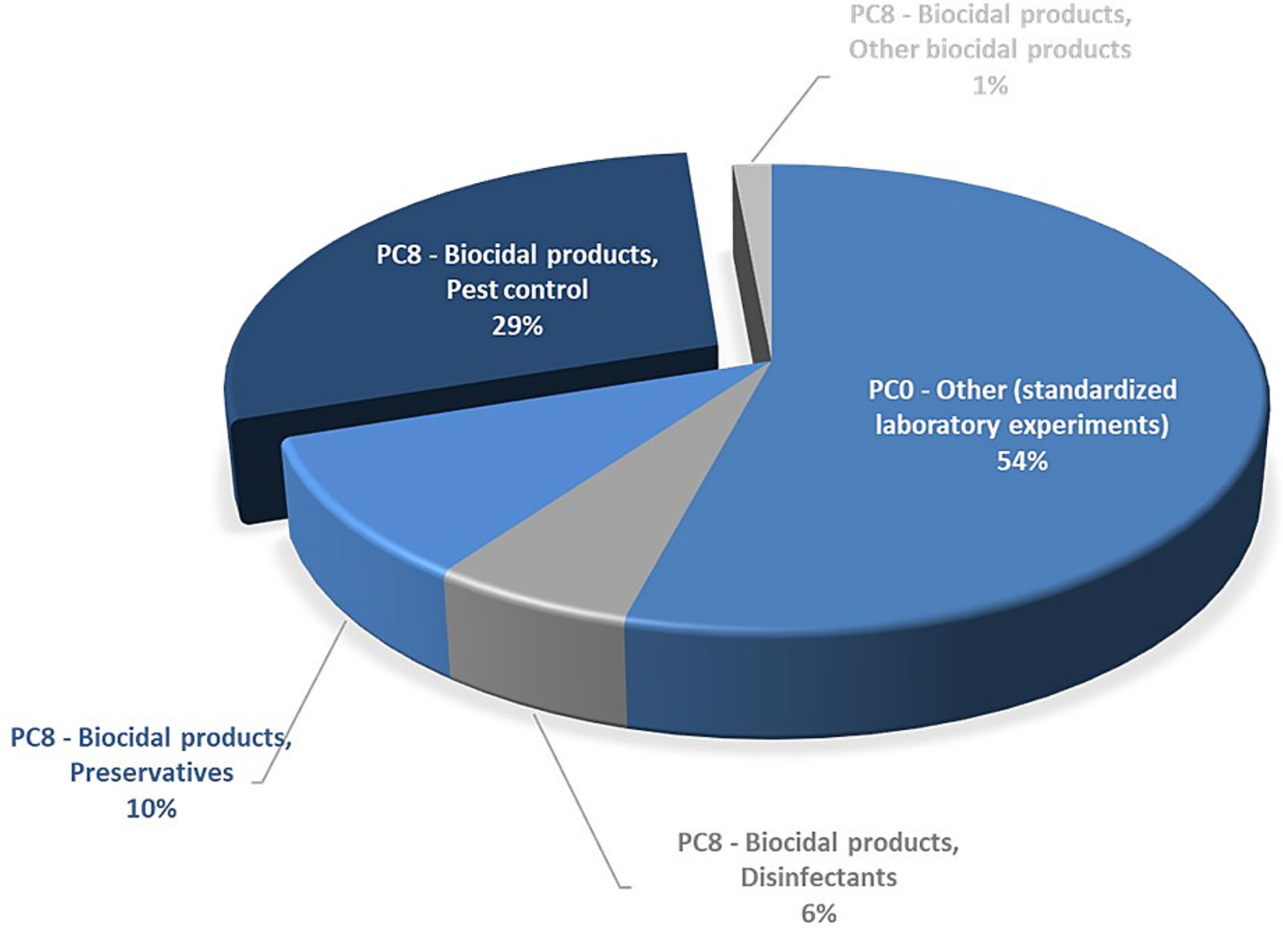
Illustration of the product categories (PCs) within the raw data originating from BAuA research projects.

The datasets of BAuA’s contribution to the raw data pool originate from seven research projects either led by or with significant contribution from BAuA. For example, the SysDEA project carried out by TNO and BPI on behalf of BAuA. Most of the dermal exposure measurements (66%) were conducted at actual workplaces, while the remaining 34% were obtained under controlled conditions in model rooms with controlled settings (see Figure 1) Two projects exclusively investigated spray applications in various areas of biocidal use, e.g., pest control or antifouling applications [F1702 (7) and F2137 (8), respectively].

One project focused on outdoor pest control (PC 8, Main Group 3), including activities such as filling, spraying, and cleaning [F 2343 (9)]. The majority of controlled-condition measurements at artificial workplaces originated from a project that encompassed powder dumping, pouring, rolling, spraying, and handling tasks [F2349 (10)]. Another project examined spraying and foaming activities for disinfection purposes [PC 8, F2366 (11)], while two further projects focused on wood impregnation and associated operations [F1809 (12) and F2053 (13), respectively]. See Table 2, Figure 1 for an overview. In addition to dermal, several projects also conducted workplace air measurements.

Nearly 2,500 summary statistics datasets were extracted by BAuA staff from scientific publications, which are also available in DermExpoDB. Relevant publication entries were sourced from the SysDEA literature database (14).

The BAuA’s contribution with regard to summary statistical datasets in DermExpoDB is based on a literature search conducted as part of the SysDEA project. The objective of this search was to identify publications containing data on dermal exposure as well as relevant background literature, particularly regarding dermal sampling and measurement techniques. The literature search included publications up to the year 2017. The Supplementary material provides a list of the references from which the DermExpoDB datasets have been extracted to date. However, the extraction of dermal exposure data from publications identified as part of the SysDEA project, as well as their coding into DermExpoDB, has not yet been completed.

Each summary statistics dataset in DermExpoDB contains exposure values derived from a specific data collective. These exposures may refer to individual body regions (e.g., hand, arm) or to the entire body, depending on how they were reported in the original publication. The body region associated with a dataset is defined according to the first source that reported it. Within these summary statistics datasets, about 30% were sampled from the hands, 33% from the body, and only about 3% from the face. 28% of the datasets provide additional information to dermal exposure such as inhalation exposure, surface contamination, biomonitoring, or oral exposure. Another 5% of the datasets reflect combined dermal exposure, for example, involving both body and face, hands and body, or hands and face.

As each dataset is defined by the first source that reported it, multiple entries in DermExpoDB may be based on the same group of sampled workers or volunteers and on the same experiments, leading to potential overlaps. For example, a single study might report statistical values for hand exposure, arm exposure, and combined hand and arm exposure. Although these entries are related, the combined exposure value (hand + arm) cannot be mathematically derived from the individual values, as only summary statistics (e.g., means, medians, standard deviations) are publicly available. To preserve the original context and integrity of the data, each reported body region is included as a separate entry in DermExpoDB, where applicable. Some summary statistics datasets may also appear partially redundant if the original source reported values in different units that cannot be converted, due to missing information such as surface size or measurement duration.

**Figure 2:**
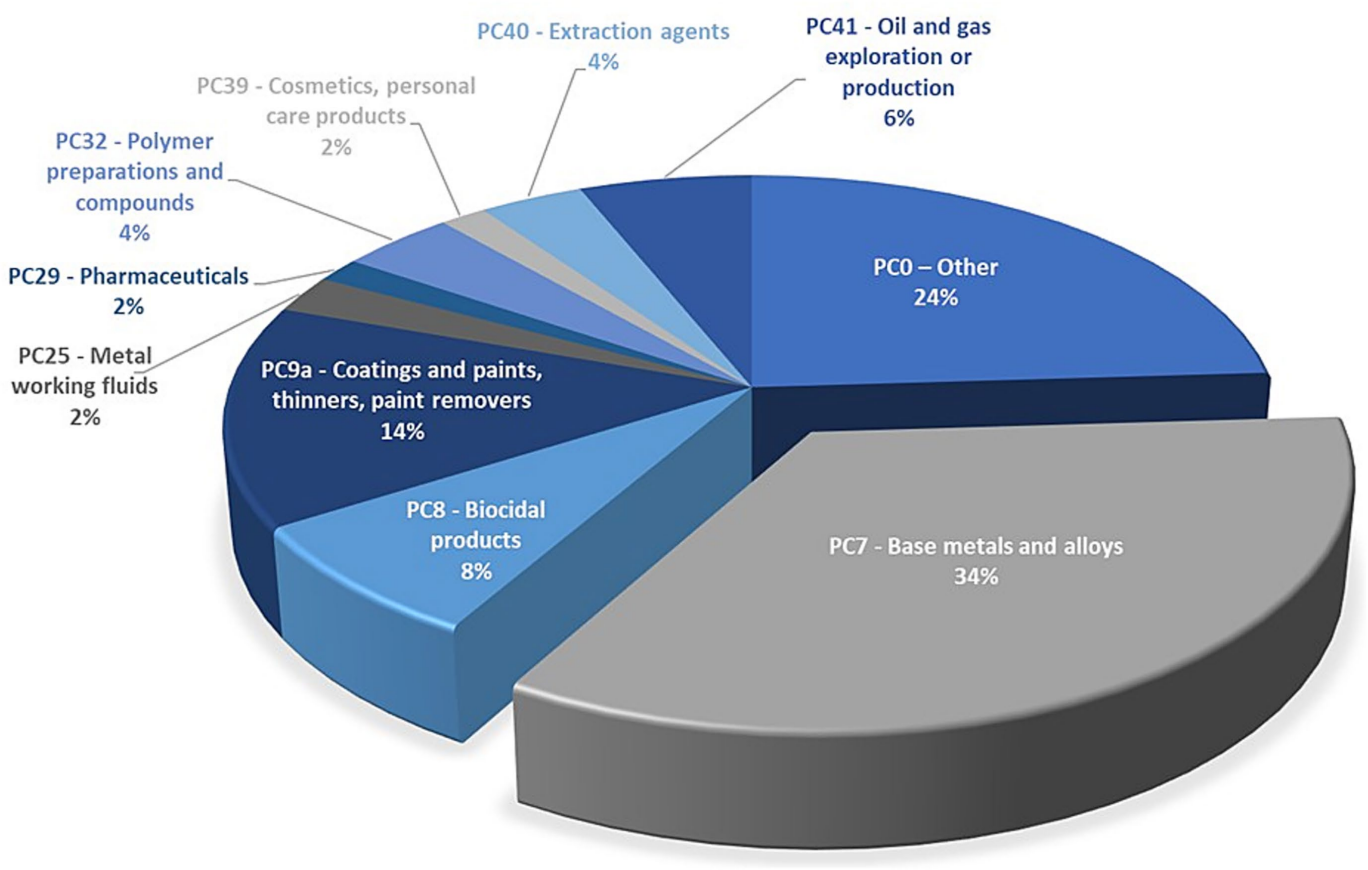
Illustration of the product categories (PCs) based on summary data from extracted scientific literature.

The most predominant product category in BAuA’s contribution to the summary statistics datasets is PC7 (base metals and alloys), accounting for 34% of all datasets (see Figure 2). This is followed by PC0—Other (primarily asphalt, asphalt paving materials, and flame retardants) with 24%, PC09a (coatings and paints, thinners, paint removers) with 14%, and PC8 (biocidal products) with 8%. Additional product categories represented—though to a lesser extent—include PC41 (oil and gas exploration or production products), PC32 (polymer preparations and compounds), PC40 (extraction agents), PC25 (metalworking fluids), PC39 (cosmetics and personal care products), and PC29 (pharmaceuticals), listed in descending order of prevalence (see Figure 2). It should be noted that the representation and significance of specific product categories may change in the future as more datasets will be added to DermExpoDB.

The majority of the summary statistics datasets (76%) originate from field studies conducted under normal working conditions. An additional 11% come from experimental, semi-experimental, or laboratory studies. The third most common study design involves the development of measurement techniques (with or without application in a field study), accounting for 6% of the datasets. Field studies under influenced work conditions (4%) and intervention field studies (2%) represent only a small portion of the available datasets.

With regard to the dermal exposure datasets, these were collected using a variety of dermal sampling techniques, particularly surrogate methods such as pads/patches, passive organic dermal (POD) samplers, whole-body techniques using Tyvek or cotton coveralls, and glove sampling. Removal techniques such as tape stripping, washing, and/or wiping were also applied. *In situ* detection methods using UV/fluorescence (e.g., video imaging and visualization systems like FIVES and VITAE) are currently only scarcely represented.

The coding of the datasets owned by TNO, EBRC and EUROMATAUX is also ongoing and will include additional dermal exposure data.

TNO’s contribution with regard to summary statistical datasets in DermExpoDB is based on, amongst others, a literature search conducted as part of the BROWSE project (15) and the Cefic LRI B16 project (16). The objective of this search was to identify publications containing occupational exposure data, with a focus on (dermal) exposure to plant protection products, but biocidal products and industrial chemicals were also included. The BROWSE database consists of over 2000 individual measurements from 140 studies and the CEFIC database consists of 106 summary measurements from 35 data sources. The checking for doublets/duplicates and coding into DermExpoDB has not yet been completed. TNO’s contribution with regard to raw datasets is based on the Cefic LRI project B20—Experimental assessment of inhalation and dermal exposure to chemicals during industrial and professional activities, including 70 dermal exposure measurements covering seven scenarios of five different PROCs.

Several data sets on zinc, lead, nickel and calcium carbonate are available from the European non-ferrous metals industry (17–27) in aggregated format (as summary statistics) which will be included in the database. In addition, EBRC has access to the associated raw data for most of the associated studies and has permission to enter these into the database. In such cases, it is foreseen to include the individual raw data in DermExpoDB.

## Discussion and conclusions

DermExpoDB was developed as a balanced compromise between compliance with intellectual property rights, consideration of data owner sensitivities and aspects of the FAIR (Findability, Accessibility, Interoperability, and Reusability) data principles. This was achieved while taking into account the practical constraints of time and financial resources, given that all project partners contributed their time and resources on their own “funding.”

In conclusion, DermExpoDB is a unique resource, which pools occupational dermal exposure data from various institutes in order to improve our understanding of, and ability to manage, occupational dermal exposure. The database currently contains already more than 3,000 datasets and will continue to grow in the future. By pooling exposure data in a structured format, users can explore exposure data across different chemicals, workplaces, protective measures, and further categories. This provides a valuable resource for more accurate scientific innovation in dermal risk assessments, advances our understanding of occupational dermal exposure to hazardous chemicals and helps to develop and refine predictive models.

### Call to action

If any occupational dermal exposure data are available for sharing, institutes or individuals are invited to get in touch via the DermExpoDB website (https://dermexpodb.baua.de/, for correspondence please contact us at DermExpoDB@baua.bund.de). The team is always seeking additional data to expand the scope of DermExpoDB and would be pleased to hear from potential contributors. Similarly, if occupational dermal exposure data are needed for scientific research or development purposes, individuals are invited to register in DermExpoDB. Registered users can access and contribute to the database, ensuring its continuous growth and improvement.

## Data Availability

Publicly available datasets were analyzed in this study. This data can be found at: https://dermexpodb.baua.de/.
