# Supplementary material for: Introducing DermExpoDB: a collaborative online database software application for occupational dermal exposure data
Source: Front Public Health. 2026 Jun 3;14:1765605. doi: 10.3389/fpubh.2026.1765605 (PMC13274560; doi:10.3389/fpubh.2026.1765605)
Supplement: Supplementary file 1 [file Data_Sheet_1.pdf]

Supplementary Material 1- References

Authors	Title	Journal	Year	Digital identifier Availability checked on 15.8.2025
Väänänen V, Hämeilä M, Kontsas H, Peltonen K, Heikkilä P	Air concentrations and urinary metabolites of polycyclic aromatic hydrocarbons among paving and remixing workers	Journal of Environmental Monitoring, 5: 739-746	2003	https://doi.org/10.1039/B304096H
Väänänen V, Hämeilä M, Kalliokoski P, Nykyri E, Heikkilä P	Dermal Exposure to Polycyclic Aromatic Hydrocarbons among Road Pavers	Annals of Occupational Hygiene, 49 (2): 167-178	2005	https://doi.org/10.1093/annhyg/meh094
Lind ML, Boman A, Sollenberg J, Johnsson S, Hagelthorn G, Meding B,	Occupational Dermal Exposure to Permanent Hair Dyes Among Hairdressers	Annals of Occupational Hygiene, 49 (6): 473-480	2005	https://doi.org/10.1093/annhyg/mei030
Liljelind IE, Eriksson KA, Nilsson LO, Jonsson IBM, Burström YI,	A method for measuring the potential dermal exposure to methyl methacrylate during two different dental technical work tasks	Journal of Environmental Monitoring, 7: 519 - 523	2005	https://doi.org/10.1039/b415207g
Chao E, Gibson R, Nylander-French L	Dermal Exposure to Jet Fuel (JP-8) in US Air Force Personnel	Annals of Occupational Hygiene, 49 (7): 639-645	2005	http://dx.doi.org/10.1093/annhyg/mei021
van Wendel de Joode B, Bierman E, Brouwer DH, Spithoven J, Kromhout H,	An assessment of dermal exposure to semi-synthetic metal working fluids by different methods to group workers for an epidemiological study on dermatitis	Journal of Occupational and Environmental Medicine, 62: 663-641	2005	https://doi.org/10.1136/oem.2004.015396
Hughson GW	An occupational hygiene assessment of dermal inorganic lead exposures in primary and intermediate user industries	Institute of Occupational Medicine (IOM) Research Report TM/04/06	2005	https://www.iom-world.org/
Hughson GW	An occupational hygiene assessment of dermal nickel exposures in primary production and primary user industries - Phase 2 Report	Institute of Occupational Medicine (IOM) Research Report TM/05/06	2005	https://www.iom-world.org/
van Wendel de Joode B, Tielemans E, Vermeulen R, Wegh H, Kromhout H	Dermal exposure assessment to benzene and toluene using charcoal cloth pads	Journal of Exposure Analysis and Enviromental Epidemiology (2005), 47-50	2005	https://doi.org/10.1038/sj.jea.7500349

Authors	Title	Journal	Year	Digital identifier Availability checked on 15.8.2025
Sleeuwenhoek A, Van Tongeren M,	Assessment of dermal exposure to inorganic lead caused by direct skin contact with lead sheet and moulded PVC profiles	Institute of Occupational Medicine (IOM) Research Report TM/06/04	2006	https://www.iom-world.org/
Veldhof RJG, Lurvink MWM, de Vreede SAF, Gijsbers JHJ, Nij ET, Brouwer DH	Dermal exposure to dust during manual transfer of powder, the effect of amount handled and dustiness	Netherlands Organization for Applied Scientific Research (TNO) report V5816	2006	https://www.iom-world.org/
Hutton S, Central Science Laboratory	Exposure to pesticides during amateur applications of home and garden products	Defra, Science Directorate	2006	https://sciencesearch.defra.gov.uk/ProjectDetails?ProjectID=13882&
Lingk W, Reifenstein H, Westphal D, Plattner E	Humanexposition bei Holzschutzmitteln	BfR Wissenschaft	2006	ISBN 3-938163-13-5
Pronk A, Vlaanderen J, Yu F, Tielemans E, Preller L, Bobeldijk I, Deddens JA, Latza U, Baur X, Heederik D	Dermal, inhalation, and internal exposure to 1,6-hexamethylene diisocyanate and its oligomers in car body repair shop workers and industrial spray painters	Journal of Occupational and Environmental Medicine, 63: 624-631	2006	http://dx.doi.org/10.1136/oem.2005.023226
Fent K, Jayaraj K, Gold A, Ball L, Nylander-French L	Tape-strip sampling for measuring dermal exposure to 1,6-hexamethylene diisocyanate	Scandinavian Journal of Work, Environment and Health, 32(3): 225-240	2006	http://dx.doi.org/10.5271/sjweh.1003
Väänänen V, Elovaara E, Nykyri E, Santonen T, Heikkilä P	Road pavers occupational exposure to asphalt containing waste plastic and tall oil pitch	Journal of Environmental Monitoring, 8: 89-99	2006	http://dx.doi.org/10.1039/b513505b
Staton I, Ma R, Evans N, Hutchinson RW, McLeod CW, Gawkrodger DJ	Dermal nickel exposure associated with coin handling and in various occupational settings: assessment using a newly developed finger immersion method	British Journal of Dermatology, 154: 658-664	2006	https://doi.org/10.1111/j.1365-2133.2006.07128.x
Cocker J, Roff M, Handley R, Frost S, Mogridge R	Efficacy of gloves used in printing: A volunteer study	HSL/2006/02	2006	https://www.hse.gov.uk/research/hsl_pdf/2006/hsl0602.pdf (not available anymore)
Berger-Preiß E, Koch W, Gerling S, Kock H, Klasen J, Hoffmann G, Appel KE	Aircraft disinsection: Exposure assessment and evaluation of a new pre-embarkation method	International Journal of Hygiene and	2006	http://dx.doi.org/10.1016/j.ijheh.2005.08.009

Authors	Title	Journal	Year	Digital identifier Availability checked on 15.8.2025
		Environmental Health, 209: 41-56		
Vermeulen R, Lan Q, Li G, et al.	Assessment of dermal exposure to benzene and toluene in shoe manufacturing by activated carbon cloth patches	Journal of Environmental Monitoring, 8: 1143-1148	2006	https://doi.org/10.1039/B608076F
Cirla P, Martinotti I, Buratti M, Fustinoni S, Campo L, Zitto E, Prandi E, Longhi O, Cavallo D, Foa V	Assessment of exposure to polycyclic aromatic hydrocarbons (PAH) in Italian asphalt workers	Journal of Occupational and Environmental Hygiene, 4(S1): 87-99	2007	https://doi.org/10.1080/15459620701354325
McClean MD, Rinehart RD, Sapkota A, Cavallari J, Herrick RF	Dermal exposure and urinary 1-Hydroxypyrene among asphalt roofing workers	Journal of Occupational and Environmental Hygiene, 4(S1): 118-126	2007	https://doi.org/10.1080/15459620701334756
McNally K, Binch S	Survey findings from a national survey of MbOCA exposure	HSL/2007/07	2007	https://www.hse.gov.uk/research/hsl_pdf/2007/hsl0707.pdf (not available anymore)
Henriks-Eckerman M, Suuronen K, Jolanki R, Riala R, Tuomi T	Determination of occupational exposure to alkanolamines in metal-working fluids	Annals of Occupational Hygiene, 51 (2): 153-160	2007	http://dx.doi.org/10.1093/annhyg/mel079
Semple S	The causative factors of dermatitis among workers exposed to metalworking fluids	Institute of Occupational Medicine (IOM) RR577	2007	https://www.hse.gov.uk/research/rrpdf/rr577.pdf (not available anymore)
Day GA, Dufresne A, Stefaniak AB, Schuler CR, Stanton ML, Miller WE, Kent MS, Deubner DC, Kreiss K, Hoover MD	Exposure Pathway Assessment at a Copper-Beryllium Alloy Facility	Annals of Occupational Hygiene, 51 (1): 67-80	2007	http://dx.doi.org/10.1093/annhyg/mel041
Christopher Y, Van Tongeren M, Cowie H, Cherrie JW	Occupational dermal exposure to heavy fuel oils	Institute of Occupational Medicine (IOM) Research Report TM/07/05	2007	https://www.iom-world.org/
Liljelind I, Michel I, Damm M, Eriksson K	Development, Evaluation and Data Acquired with a Tape-Stripping Technique for Measuring Dermal Exposure to Budesonide at a Pharmaceutical Manufacturing Site	Annals of Occupational Hygiene, 51 (4): 407-413	2007	http://dx.doi.org/10.1093/annhyg/mem015

Authors	Title	Journal	Year	Digital identifier Availability checked on 15.8.2025
Edwards JW, Lee SG, Heath LM, Pisaniello DL	Worker exposure and a risk assessment of Malathion and Fenthion used in the control of Mediterranean fruit fly in South Australia	Environmental Research, 103: 38-45	2006	http://dx.doi.org/10.1016/j.envres.2006.06.001
Chang FK, Chen ML, Cheng SF, Shih TS, Mao IF	Dermal Absorption of Solvents as a Major Source of Exposure Among Shipyard Spray Painters	Journal of Occupational and Environmental Medicine 49(4): 430-436	2007	http://dx.doi.org/10.1097/JOM.0b013e31803b94ac
Links IHM, Van der Jagt KE, Christopher Y, Lurvink M, Schinkel J, Tielemans E, van Hemmen JJ	Occupational Exposure During Application and Removal of Antifouling Paints	Annals of Occupational Hygiene, 51 (2): 207-218	2007	http://dx.doi.org/10.1093/annhyg/mel074
Mielke H, Schneider H, Westphal D, Uhlig S, Simon K, Antoni S, Plattner E	Humanexposition bei Holzschutzmitteln Neufassung der Gesamtauswertung von Haupt- und Ergänzungsstudie	BfR Wissenschaft	2008	ISBN 3-938163-38-0
Fent K, Jayaraj K, Ball L, Nylander-French L	Quantitative monitoring of dermal and inhalation exposures to 1,6-hexamethylene diioscyanate monomer and oligomers	Journal of Environmental Monitoring, 10: 500-507	2008	https://doi.org/10.1039/B715605G
Boeniger M,Neumeister C,Booth-Jones A	Sampling and analytical method development and hand wipe measurements of dermal exposures to polycyclic aromatic hydrocarbons	Journal of Occupational and Environmental Hygiene, 5:417-425	2008	https://doi.org/10.1080/15459620802111319
Bello D, Redlich CA, Stowe MH, Sparer J, Woskie SR, Streicher RP, Hosgood HD, Liu YC	Skin exposure to aliphatic polyisocyanates in the auto body repair and refinishing industry: II. A quantitative assessment	Annals of Occupational Hygiene, 52 (2): 117-124	2008	http://dx.doi.org/10.1093/annhyg/mem066
Liden C, Skare L, Nise G, Vahter M	Deposition of nickel, chromium, and cobalt on the skin in some occupations assessment by acid wipe sampling	Contact Dermatitis, 58: 347-354	2008	https://doi.org/10.1111/j.1600-0536.2008.01326.x
Eriksson K, Hagström K, Axelsson S, Nylander-French L	Tape-stripping as a method for measuring dermal exposure to resin acids during wood pellet production	Journal of Environmental Monitoring, 10: 345-352	2008	http://dx.doi.org/10.1039/b719152a
Chen M, Tsai P, Wang Y	Assessing inhalatory and dermal exposures and their resultant health-risks	Environment International, 34: 971-975	2008	http://dx.doi.org/10.1016/j.envint.2008.02.008

Authors	Title	Journal	Year	Digital identifier Availability checked on 15.8.2025
	for workers exposed to polycyclic aromatic hydrocarbons (PAHs) contained in oil mists in a fastener manufacturing industry			
Fent K, Gaines LGT, Thomasen J, Flack S, Ding K, Herring A, Whittaker S., Nylander-French L	Quantification and Statistical Modeling - Part I: Breathing-Zone Concentrations of Monomeric and Polymeric 1,6-Hexamethylene Diisocyanate	Annals of Occupational Hygiene, 53 (7): 677-689	2009	https://doi.org/10.1093/annhyg/mep046
Fent K, Gaines LGT, Flack S, Ding K, Herring A, Whittaker S., Nylander-French L, Thomasen J	Quantification and Statistical Modeling - Part II: Dermal Concentrations of Monomeric and Polymeric 1,6-Hexamethylene Diisocyanate	Annals of Occupational Hygiene, 53 (7): 691-702	2009	https://doi.org/10.1093/annhyg/mep048
Virji MA, Woskie SR, Pepper LD	Skin and Surface Lead Contamination, Hygiene Programs, and Work Practices of Bridge Surface Preparation and Painting Contractors	Journal of Occupational and Environmental Hygiene, 6 (2): 131-142	2008	https://doi.org/10.1080/15459620802656636
Virji MA, Woskie SR, Pepper LD	Task-Based Lead Exposures and Work Site Characteristics of Bridge Surface Preparation and Painting Contractors	Journal of Occupational and Environmental Hygiene, 6:2, 99-112	2008	https://doi.org/10.1080/15459620802615772
Shih TS, Kuo YC, Liang RH, Liou SH, Chang HY, Chou TC	Assessment of Airborne and Dermal Exposure to 2-Ethoxyethyl Acetate in an Occupational Environment	American Journal of Industrial Medicine, 52: 654-661	2009	http://dx.doi.org/10.1002/ajim.20709
Mäkinen MS, Mäkinen MR, Koistinen J, Pasanen AL, Pasanen P, Kalliokoski P, Korpi A	Respiratory and dermal exposure to organophosphorus flame retardants and tetrabromobisphenol A at five work environments	Environmental Science and Technology, 43 (3): 941-947	2009	http://dx.doi.org/10.1021/es802593t
Liljelind IE, Hagenbjörk-Gustafsson A, Nilsson LO	Potential dermal exposure to methyl methacrylate among dental technicians, variability and determinants in a field study	Journal of Environmental Monitoring, 11: 160-165	2009	https://doi.org/10.1039/b810355k
Christopher Y, Van Tongeren M	Occupational dermal exposure to heavy fuel oil - Part II	Institute of Occupational Medicine (IOM) Research Report TM/08/05	2009	https://www.iom-world.org/

Authors	Title	Journal	Year	Digital identifier Availability checked on 15.8.2025
Stefaniak AB, Virji MA, Day GA	Characterization of exposures among cemented tungsten carbide workers. Part I: Size-fractionated exposures to airborne cobalt and tungsten particles	Journal of Exposure Science and Environmental Epidemiology, 19: 475-491	2009	https://doi.org/10.1038/jes.2008.37
Day GA, Virji MA, Stefaniak AB	Characterization of exposures among cemented tungsten carbide workers. Part II: Assessment of surface contamination and skin exposures to cobalt, chromium and nickel	Journal of Exposure Science and Environmental Epidemiology, 19: 423-434	2009	http://dx.doi.org/10.1038/jes.2008.33
Gaines L, Fent K, Flack S, Thomasen J, Ball L, Richardson D, Ding K, Whittaker S, Nylander-French L	Urine 1,6-Hexamethylene Diamine (HDA) Levels Among Workers Exposed to 1,6-Hexamethylene Diisocyanate (HDI)	Annals of Occupational Hygiene, 54 (6): 678-691	2010	http://dx.doi.org/10.1093/annhyg/meq041
Fustinoni S, Campo L, Cirla P, Martinotti I, Buratti M, Longhi O, Foa V, Bertazzi PA	Dermal epxosure to polycyclic aromatic hydrocarbons in asphalt workers	Journal of Occupational and Environmental Medicine, 67: 456-463	2010	http://dx.doi.org/10.1136/oem.2009.050344
Keen C, Coldwell M, McNally K, Baldwin P, McAlinden J	Occupational exposure to MbOCA (4,4-methylene-bis-ortho-chloroaniline) and isocyanates in polyurethane manufacture	Health and Safety Executive 2010	2010	https://www.hse.gov.uk/research/rrpdf/rr828.pdf (not available anymore)
Julander A, Skare L, Mulder M, Grandér M, Vahter M, Lidén C	Skin Deposition of Nickel, Cobalt, and Chromium in Production of Gas Turbines and Space Propulsion Components	Annals of Occupational Hygiene, 54 (3): 340-350	2010	https://doi.org/10.1093/annhyg/meq002
Liljelind I, Norberg C, Egelrud L, Westberg H, Eriksson K, Nylander-French L	Dermal and Inhalation Exposure to Methylene Bisphenyl Isocyanate (MDI) in Iron Foundry Workers	Annals of Occupational Hygiene, 54 (1): 31-40	2010	http://dx.doi.org/10.1093/annhyg/mep067
Du Plessis JL, Eloff FC, Badenhorst CJ, Olivier J, Laubscher PJ, Van Aarde MN, Franken A	Assessment of Dermal Exposure and Skin Condition of Workers Exposed to Nickel at a South African Base Metal Refinery	Annals of Occupational Hygiene, 54 (1): 23-30	2010	http://dx.doi.org/10.1093/annhyg/mep080
Olsen LD, Snawder JE, Kriech AJ, Osborn LV	Development of a 5-Layer Passive Organic Dermal (POD) Sampler	Polycyclic Aromatic Compounds, 31: 154-172	2011	https://doi.org/10.1080/10406638.2011.581262
De Vries T, Bello D, Stowe M, Harari H, Slade M, Redlich C	Transferability of aliphatic isocyanates from recently applied paints to the skin of auto body shop workers	Journal of Occupational and Environmental Hygiene, 9: 699-711	2012	https://doi.org/10.1080/15459624.2012.728893

Authors	Title	Journal	Year	Digital identifier Availability checked on 15.8.2025
Cavallari J, Osborn L, Snawder J, Kriech A, Olsen L, Herrick RF, McClean MD	Predictors of Dermal Exposures to Polycyclic Aromatic Compounds Among Hot-Mix Asphalt Paving Workers	Annals of Occupational Hygiene, 56 (2): 125-137	2012	https://doi.org/10.1093/annhyg/mer108
Galea KS, Davis A, Todd D, McGonagle C, MacCalman L, Cherrie JW	Determination of the potential for dermal exposure from transfer of lubricants and fuels by consumers	Institute of Occupational Medicine (IOM) Research Report TM/13/03	2013	https://www.iom-world.org/
Fent K, Eisenberg J, Evans D, Sammons D, Robertson S, Striley CAF, Snawder J, Mueller C, Kochenderfer V, Pleil J, Stiegel M, Horn G	Evaluation of Dermal Exposure to Polycyclic Aromatic Hydrocarbons in Fire Fighters	Health Hazard Evaluation Programm, Report No. 2010-0156-3196	2013	https://www.cdc.gov/niosh/hhe/reports/pdfs/2010-0156-3196.pdf
Keen C, Tan E, McAlinden J, Woolgar P, Smith P	Exposure to hexavalent chromium, nickel and cadmium compounds in the electroplating industry	Health and Safety Executive 2013	2013	https://www.hse.gov.uk/research/rrpdf/rr963.pdf (not available anymore)
Harari H, Bello D, Woskie S, Redlich C	Development of an Interception Glove Sampler for Skin Exposures to Aromatic Isocyanates	Annals of Occupational Hygiene, 60 (9) 1092-1103	2016	https://doi.org/10.1093/annhyg/mew052
Klasson M, Lindberg M, Bryngelsson IL, Arvidsson H, Pettersson C, Husby B, Westberg H	Biological monitoring of dermal and air exposure to cobalt at a Swedish hard metal production plant: does dermal exposure contribute to uptake?	Contact Dermatitis, 77: 201-207	2017	https://doi.org/10.1111/cod.12790
Thomasen JM, Fent KW, Nylander-French LA	Development of a Sampling Patch to Measure Dermal Exposures to Monomeric and Polymeric 1,6-Hexamethylene Diisocyanate: A Pilot Study	Journal of Occupational and Environmental Hygiene, 8 (12): 709-717	2011	https://doi.org/10.1080/15459624.2011.626744

Supplementary Material 2 – DermExpoDB database structure

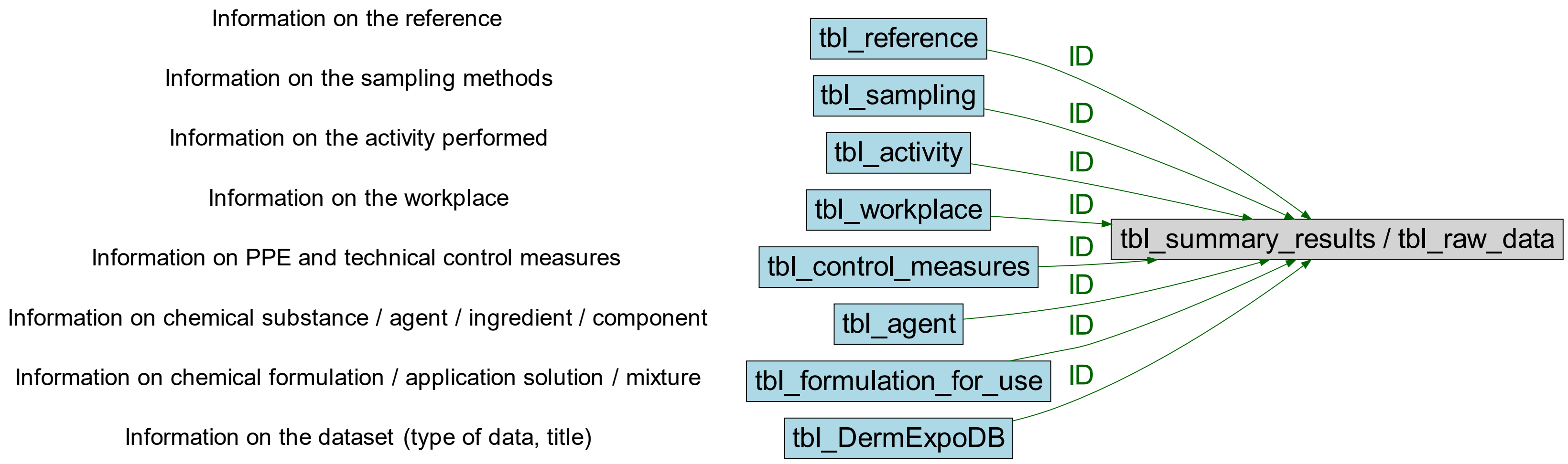

Supplementary Material 3 – database scheme: ER diagram for raw data

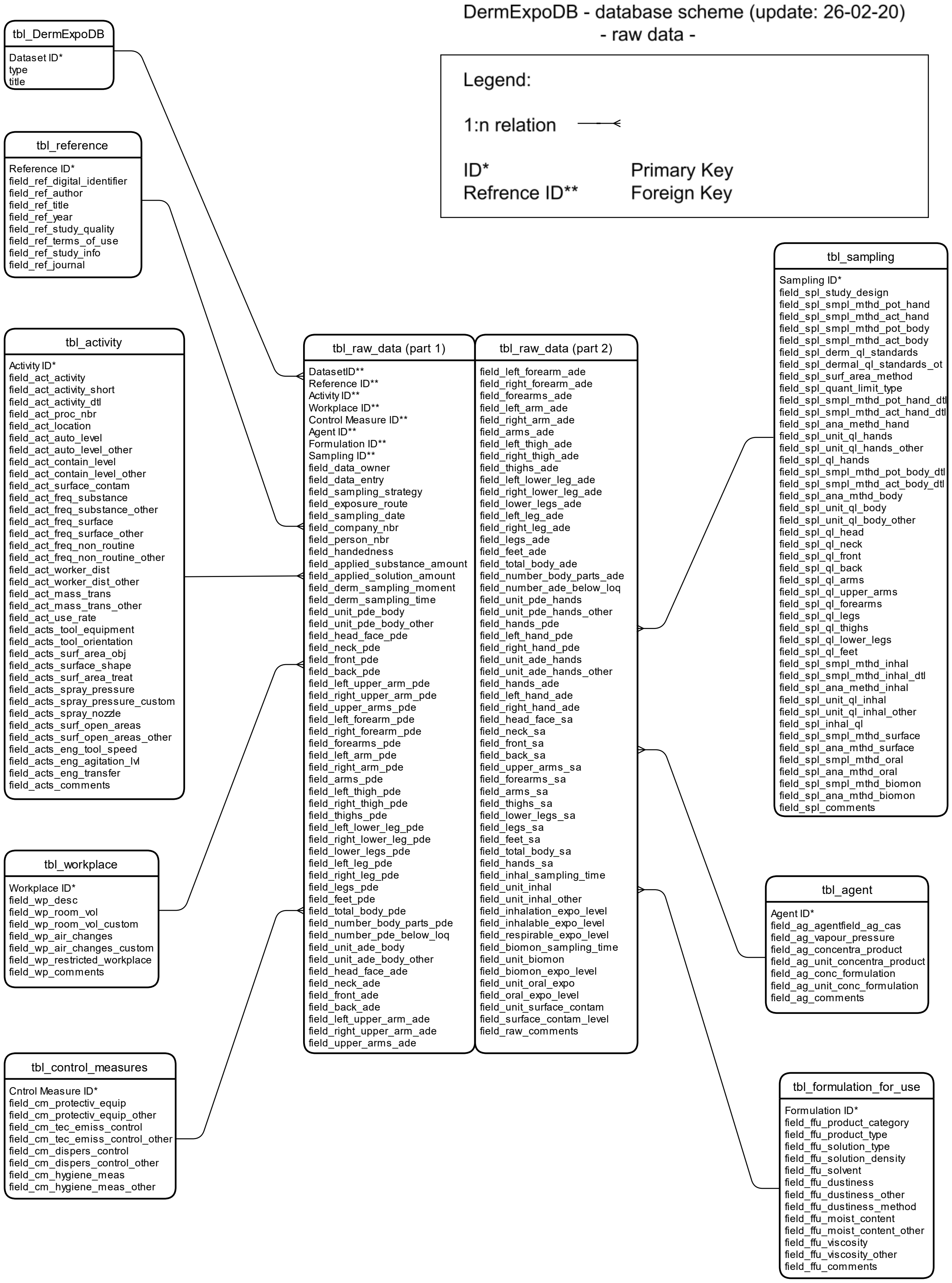

Supplementary Material 4 – data field list for raw data

Machine name	Field name	Field input description
**type**	**Type of data**	*Specify the type of data that is uploaded in DermExpoDB.*
**title**	**Dataset title**	*Specify a unique title for each data record.*
**field_ref_digital_identifier**	**Digital identifier**	*Include DOI or ISBN or web address if the data is publically available. If it is not publicly available, insert "unpublished".*
**field_ref_author**	**Author(s) of study**	*Name the author(s) which originally published the data. Preferred format is: Fritz DH, Mayer JJ.*
**field_ref_title**	**Reference title**	*Provide the title of the report/publication in which the data was published.*
**field_ref_year**	**Year**	*Specify the year in which study or data was published.*
**field_ref_study_quality**	**Study quality**	*Judge the quality of the study.*
**field_ref_terms_of_use**	**Terms of use of data**	*Decide whether your data is flagged as confidential or non-confidential data upon upload in the database software application. Outside parties can only request access to non-confidential data. Data indicated as being confidential upon upload may not be used without the approval by the Steering Committee by any user.*
field_ref_study_info	**Abstract / background information of study**	*Submit an abstract or executive summary of the study.*
field_ref_journal	**Journal / Report**	*Identify the journal or publisher that published the data. Preferred format is journal name, volume (issue): pages or publisher identifier.*
**field_act_activity**	**Activity**	*Provide the activity category. It is possible to select more than one element from the predefined list.*
**field_act_activity_short**	**Activity - Short description**	*Provide a brief description of the activity performed during the measurement.*
**field_act_activity_dtl**	**Activity - details**	*Give a detailed description including all relevant contextual information of the activity, work tasks or processes performed during the measurement.*
**field_act_proc_nbr**	**PROC number & PROC description**	*Select the PROC number / PROC description that is relevant for the major activity, i.e. which dominates the exposure. It is possible to select more than one element from the predefined list.*
**field_act_location**	**Location**	*Distinguish whether an indoor or outdoor application or both was carried out.*

field_act_auto_level	**Level of automation**	*Record the degree of automation of the process. Predefined categories are available as well as a free text option.*
field_act_auto_level_other	**Level of automation - Free text**	
field_act_contain_level	**Level of containment**	*Record the degree of enclosure of an activity or process. Predefined categories are available as well as a free text option.*
field_act_contain_level_other	**Level of containment - Free text**	
field_act_surface_contam	**Surface contamination**	*Describe visible, measured or judged surface contamination (contaminated surfaces) with which the worker comes into contact during the activity. Relevent information are i.e. level of contamination or % surface area contaminated. Reference to available quantitative, measured surface contamination data.*
field_act_freq_substance	**Contact frequency with chemical substance / product / formulation**	*Provide recorded information or own judgement on frequency of routine contact with the chemical product / formulation / application solution / mixture / substance / agent / ingredient / component e.g. direct contact by touching solid objects or immersion of body parts in chemical. Predefined categories are available as well as a free text option.*
field_act_freq_substance_other	**Contact frequency with chemical substance / product / formulation - Free text**	
field_act_freq_surface	**Contact frequency with contaminated surfaces**	*Provide recorded information or own judgement on frequency of routine contact with equipment, tools, control panels or or other contaminated objects. Predefined categories are available as well as a free text option.*
field_act_freq_surface_other	**Contact frequency with contaminated surfaces - Free text**	
field_act_freq_non_routine	**Contact frequency due to non-routine activities**	*Provide recorded information or own judgement on frequency of non-routine contact such as contact during trouble shooting, maintenance or repairs (e.g. unblocking nozzles). Predefined categories are available as well as a free text option.*

field_act_freq_non_routine_other	**Contact frequency due to non-routine activities - Free text**	
field_act_worker_dist	**Distance worker-source**	*Estimate the distance between worker and emission source. Predefined categories are available as well as a free text option.*
field_act_worker_dist_other	**Distance worker-source - Free text**	
field_act_mass_trans	**Relevant mass transport processes**	*Specify the mass transport processes that were observed during the measurement. For description of dermal mass transport processes: Schneider T, Vermeulen R, Brouwer DH, Cherrie JW, Kromhout H, Fogh CL. Conceptual model for assessment of dermal exposure. Occup Environ Med. 1999 Nov;56(11):765-73. doi: 10.1136/oem.56.11.765. PMID: 10658563; PMCID: PMC1757678. Predefined categories are available as well as a free text option. It is possible to select more than one element from the predefined list.*
field_act_mass_trans_other	**Relevant mass transport processes - Free text**	
field_act_use_rate	**Product use rate / scale**	*Specify the (recommended) use rate for the handled product (preferably in kg/min for solids or L/min for liquids). Please also include the unit.*
field_acts_tool_equipment	**Activities with tools: Equipment**	*Only relevant for activities where tools are used: Give a detailed description of all equipment that was used during the measurement, i.e. spray equipment or long-handle brush.*
field_acts_tool_orientation	**Activities with tools: Orientation of work**	*Only relevant for activities where tools are used: Indicate the direction or orientation in which a product is applied in relation to worker. Choose from predefined categories.*
field_acts_surf_area_obj	**Activities with treated objects: Area of treated objects**	*Only relevant for activities where objects are treated: Specify the surface area of the object (in m²) that was treated during the measurement.*
field_acts_surface_shape	**Activities with treated objects: Surface shape of treated objects**	*Only relevant for activities where objects are treated: Outline the shape of the object treated e.g. by spraying. Choose from predefined categories.*
field_acts_surf_area_treat	**Activities with treated surfaces:**	*Only relevant for activities where (flat) surface areas are treated: Specify the area of the (flat) surface (in m²) that was treated during the measurement.*

	**Area of treated (flat) surface**	
field_acts_spray_pressure	**Spraying: Spray pressure**	*Only relevant for spray activities: Specify the spray pressure (in bar) that was set during the measurement on the spraying device. Predefined categories are available as well as a numerical input option.*
field_acts_spray_pressure_custom	**Spraying: Spray pressure - Numerical value**	
field_acts_spray_nozzle	**Spraying: Spray nozzle**	*Only relevant for spray activities: Give information on the spray nozzle (i.e. name of spray nozzle, spray nozzle area, spray nozzle angle) attached to the spraying device that was used during the measurement.*
field_acts_surf_open_areas	**Activities with open surfaces: Area of open chemical substance / product / formulation**	*Only relevant for activities with open chemical substance / agent / ingredient / component / product / formulation / application solution / mixture surface: Enter the area of the open surface area (in m²), for example if the chemical substance / agent / ingredient/ component / product / formulation / application solution / mixture is stored in an open container, jar or vessel. Predefined categories are available as well as a numerical input option.*
field_acts_surf_open_areas_other	**Activities with open surfaces: Area of open chemical substance / product / formulation - Numerical value**	
field_acts_eng_tool_speed	**Activities with energy input: Tool speed**	*Only relevant for activities with energy input: Specify the (rotational) speed of the tool used during the measurement which can lead to an increased release of a chemical substance / agent / ingredient / component from a tool (such as spattering from rollers).*
field_acts_eng_agitation_lvl	**Activities with energy input: Agitation level**	*Only relevant for activities with energy input: Provide the level of agitation of a liquid during an activity (e.g. mixing). Choose from predefined categories.*
field_acts_eng_transfer	**Activities with energy input: Transfer type**	*Only relevant for transfer of liquids and solids: Outline the type of technique applied to transfer liquids or solids. Choose from predefined categories.*

field_acts_comments	**Other comments on activity**	*Address any other information that may be relevant and related to the activities performed during the measurement.*
**field_wp_desc**	**Workplace description**	*Provide a comprehensive description of the workplace where the measurement took place.*
field_wp_room_vol	**Indoor room volume**	*If applicable, specify the indoor air volume. Predefined categories are available as well as a numerical input option.*
field_wp_room_vol_custom	**Indoor room volume - Numerical value**	
field_wp_air_changes	**Indoor air changes / outdoors wind speed**	*For indoor applications, provide the ventilation rate in the room in which the measurement was performed. For outdoor applications, provide the wind speed. Predefined categories are available as well as a numerical input option.*
field_wp_air_changes_custom	**Indoor air changes / outdoors wind speed - Numerical value**	
field_wp_restricted_workplace	**Restricted workplace**	*Indicate whether work was performed in a restricted workspace with an increased risk of skin contact, e.g. low hanging crop, spaces entered on the knees or similar. Choose from predefined categories.*
field_wp_comments	**Other comments on workplace**	*Address any other information that may be relevant and related to the workplace where the measurement was performed.*
**field_cm_protectiv_equip**	**Personal Protective Equipment**	*Provide information on the personal protective equipment (PPE) used during the measurement. It is possible to select more than one element from the predefined list.*
field_cm_protectiv_equip_other	**Personal Protective Equipment - Free text**	
field_cm_tec_emiss_control	**Technical emission control at source**	*Describe any technical control measure at the source that was present during the measurement. Predefined categories are available as well as a free text option. It is possible to select more than one element from the predefined list.*
field_cm_tec_emiss_control_other	**Technical emission control at source - Free text**	

field_cm_dispers_control	**Dispersion control away from source**	*Describe any controls related to controlling the dispersion of air contaminants in the work environment, e.g. improved general ventilation that was present during the measurement. Predefined categories are available as well as a free text option. It is possible to select more than one element from the predefined list.*
field_cm_dispers_control_other	**Dispersion control away from source – Free text**	
field_cm_hygiene_meas	**Hygiene measures**	*Describe any hygiene measures that that have been implemented in the workplace. Predefined categories are available as well as a free text option. It is possible to select more than one element from the predefined list.*
field_cm_hygiene_meas_other	**Hygiene measures - free text**	
**field_ag_agent**	**Chemical substance / agent / ingredient / component**	*Indicate the name of the chemical substance / agent / ingredient / component to which the measured exposure refers. If more than one chemical substance / agent / ingredient / component has been measured for a given exposure situation, indicate only the chemical substance / agent / ingredient / component to which the exposure value of the data record refers to. If the measured exposure refers to a chemical product / formulation / application solution / mixture, e.g. heavy fuel oils (HFO), and napthalene was measured as an indicator, indicate accordingly, e.g. "heavy fuel oils (HFO) (napthalene as marker for exposure to HFO)".*
**field_ag_cas**	**CAS**	*Provide the CAS (Chemical Abstracts Service) number of the chemical of interest. In case a CAS is not assignable, e.g. because the measured exposure relates to a chemical product, insert „not assignable“.*
**field_ag_vapour_pressure**	**Vapour pressure (VP) of chemical substance / agent / ingredient / component**	*Provide the vapour pressure of the substance at 20 degrees celsius. Note in the data field „Other comments on chemical substance / agent / ingredient / component“ in case the inserted vapour pressure relates to a temperature other than 20 degrees celsius. If you want to indicate that the substance is non-volatile without specifying the vapor pressure further, this is stated by inserting "-1". If you can't assign a vapour pressure, because you can't retrieve it from publication or a sum of different substance was measured, this is stated by inserting "-2".*

field_ag_concentra_product	**Concentration of chemical substance / agent / ingredient / component in chemical product**	*Indicate the concentration of the chemical substance / agent / ingredient / component in the chemical product. The data field accept numerical input only. If the concentration information is only available in text form, e.g. if a concentration range is to be specified (e.g. 0.5 - 5 % or mean concentration: 5 % +/- 2%), then enter the available information on the concentration in the data field "Other comments on chemical substance / agent / ingredient / component".*
field_ag_unit_concentra_product	**Unit concentration of chemical substance / agent / ingredient / component in chemical product**	*Outline the unit in which the concentration of the chemical substance / agent / ingredient / component in the used chemical product is inserted in DermExpoDB, i.e. g/l, g/kg, weight-%, volume-%.*
field_ag_concentra_formulation	**Concentration of chemical substance in chemical formulation / application solution / mixture**	*Indicate the concentration of the chemical substance / agent / ingredient / component in the chemical formulation / application solution / mixture. If the pure chemical product was used during the measurement, this data field can either remain empty or should match the entry in the data field "Concentration of the chemical substance / agent / ingredient / component in chemical product". The data field accept numerical input only. If the concentration information is only available in text form, e.g. if a concentration range is to be specified (e.g. 0.5 - 5 % or mean concentration: 5 % +/- 2%), enter the available information on the concentration in the data field "Other comments on chemical substance / agent / ingredient / component".*
field_ag_unit_conc_formulation	**Unit concentration of chemical substance in chemical formulation / application solution / mixture**	*Outline the unit in which the concentration of the chemical substance / agent / ingredient / component in the used chemical formulation / application solution / mixture is uploaded in DermExpoDB, i.e. g/l, g/kg, weight-%, volume-%.*
field_ag_comments	**Other comments on chemical substance / agent / ingredient / component**	*Address any other information that may be relevant and related to the chemical substance / agent / ingredient / component measured during the measurement.*
**field_ffu_product_category**	**Product category**	*Describe the appearance of the chemical product used in the measurement as it was prior to use or processing. For example, is the product a liquid concentrate, a solid object, a powder, a paste etc. Choose from predefined categories.*

**field_ffu_product_type**	**Product type**	*Describe the appearance of the chemical product used in the measurement as it was prior to use or processing. For example, is the product a liquid concentrate, a solid object, a powder, a paste etc. Choose from predefined categories. It is possible to select more than one element from the predefined list.*
**field_ffu_solution_type**	**Chemical formulation / application solution / mixture type**	*Describe the appearance of the chemical formulation / application solution / mixture during use or processing. For example is the pure product handled or a treated object or a dilution of liquid concentrate etc. Choose from predefined categories. It is possible to select more than one element from the predefined list.*
field_ffu_solution_density	**Density of applied chemical product / formulation / application solution / mixture**	*Specify the density of the applied chemical product / formulation / application solution / mixture in g/L. The data field accept numerical input only. If the density information is only available in text form, e.g. if a density range is to be specified (e.g. 790-900 g/l), enter the available information on the density in the data field "Other comments on the chemical substance / agent / ingredient / component".*
field_ffu_solvent	**Solvent**	*If applicable, specify the name of the (primary) substance(s) / agent(s) / ingredient(s) / component(s) that was / were used as solvent(s) in the product.*
field_ffu_dustiness	**Dustiness**	Only relevant for solid products: Specify the dustiness of the product / material. Predefined categories are available as well as a free text option.
field_ffu_dustiness_other	**Dustiness - Free text**	
field_ffu_dustiness_method	**Dustiness test method**	Only relevant for solid products: Describe the method used to determine the dustiness of a product / material including all relevant details.
field_ffu_moist_content	**Moisture content**	Only relevant for solid products: Indicate the moisture content of the chemical product / material. This does not apply to wetting during the activity. Predefined categories are available as well as a free text option.
field_ffu_moist_content_other	**Moisture content - Free text**	
field_ffu_viscosity	**Viscosity**	*Only relevant for liquid products: Indicate the viscosity of the used chemical product / formulation / application solution / mixture. Predefined categories are available as well as a free text option.*
field_ffu_viscosity_other	**Viscosity - Free text**	

field_ffu_comments	**Other comments on chemical product / formulation / application solution / mixture**	*Address any other information that may be relevant and related to the chemical product / formulation / application solution / mixture used during the measurement.*
**field_spl_study_design**	**Study design**	*Specify the type of study (i.e. field, intervention, laboratory/experimental study) and whether the study was conducted under normal or influenced working conditions. If the study consisted of several different parts, e.g. a laboratory study followed by workplace measurements, then only the type of study relevant to the measured data at hand should be indicated. Choose from predefined categories.*
**field_spl_smpl_mthd_pot_hand**	**Sampling method potential dermal hand exposure**	*Specify the sampling method used to measure the specified exposure. Choose from predefined categories. Familiarize yourself with the definitions for potential and actual exposure as well as body and hand exposure outlined in "A guide to insert and manage content in DermExpoDB".*
**field_spl_smpl_mthd_act_hand**	**Sampling method actual dermal hand exposure**	*Specify the sampling method used to measure the specified exposure. Choose from predefined categories. Familiarize yourself with the definitions for potential and actual exposure as well as body and hand exposure outlined in "A guide to insert and manage content in DermExpoDB".*
**field_spl_smpl_mthd_pot_body**	**Sampling method potential dermal body exposure**	*Specify the sampling method used to measure the specified exposure. Choose from predefined categories. Familiarize yourself with the definitions for potential and actual exposure as well as body and hand exposure outlined in "A guide to insert and manage content in DermExpoDB".*
**field_spl_smpl_mthd_act_body**	**Sampling method actual dermal body exposure**	*Specify the sampling method used to measure the specified exposure. Choose from predefined categories. Familiarize yourself with the definitions for potential and actual exposure as well as body and hand exposure outlined in "A guide to insert and manage content in DermExpoDB".*
field_spl_derm_ql_standards	**Quality standard for dermal sampling**	*If applicable, indicate the quality standards according to which the study was carried out. This can be, for example, a standard or a reference to a publication or a previously performed study. Predefined categories are available as well as a free text option.*
field_spl_dermal_ql_standards_ot	**Quality standard for dermal sampling - Free text**	

field_spl_surf_area_method	**Method to determine the (sampler or body part) surface area**	*Outline the methodology used to determine the surface area of the samplers for the various body parts. If the method is standardized or published in a peer-reviewed publication, a reference to the standard or publication is sufficient. When the details of the methodology are not standardized or the surface area of the samplers or the methodology used to determine them are unique to the study, a detailed description of the method should be provided (e.g. by weighing, reference to standard body areas). For summary statistics data, the surface areas of all relevant samplers (e.g. samplers for the hands and different body parts) should be provided here. For raw data, individual data fields for the different samplers exist and thus do not need to be stated here. If the methods for hand and body exposure are different, both must be entered in this data field.*
field_spl_quant_limit_type	**Type of quantification limit**	*Specify any quantification limit estimated in the study, e.g. limit of detection (LOD), limit of quantification (LOQ), recovery rate or other for the analytical and / or sampling methods.*
field_spl_smpl_mthd_pot_hand_dtl	**Sampling method potential dermal hand exposure - details**	*Provide all relevant details for the method used to sample the specified exposure. This thereby includes all relevant information on transport and storage. Relevant information may be number of samples, size of patches, position of the patches, sampling material used, details on impregnation of patches, suppliers etc. Familiarise yourself with the definitions of body and hand exposure as well as potential and actual exposure presented in "A guide to insert and manage content in DermExpoDB".*
field_spl_smpl_mthd_act_hand_dtl	**Sampling method actual dermal hand exposure - details**	*Provide all relevant details for the method used to sample the specified exposure. This thereby includes all relevant information on transport and storage. Relevant information may be number of samples, size of patches, position of the patches, sampling material used, details on impregnation of patches, suppliers etc. Familiarise yourself with the definitions of body and hand exposure as well as potential and actual exposure presented in "A guide to insert and manage content in DermExpoDB".*
field_spl_ana_methd_hand	**Analytical method dermal hand exposure**	*Provide all relevant details of the analytical method used to determine the specified exposure. This thereby includes all relevant information from the point at which the samples are analyzed in the laboratory or on site. Familiarise yourself with the definitions of body and hand exposure presented in "A guide to insert and manage content in DermExpoDB".*
field_spl_unit_ql_hands	**Unit QL hands**	*Outline the units in which the quantification limits (QL) for the specified sample is inserted in DermExpoDB. Familiarize yourself with the definitions for body and hand exposure outlined in the guide. Predefined categories are available as well as a free text option.*
field_spl_unit_ql_hands_other	**Unit QL hands - Free text**	

field_spl_ql_hands	**Hands QL**	*Provide the quantification limit(s) (QL) for the specified exposure measurement in the unit specified in the data field „Unit QL hands" or „Unit QL hands - Free text".*
field_spl_smpl_mthd_pot_body_dtl	**Sampling method potential dermal body exposure - details**	*Provide all relevant details for the method used to sample the specified exposure. This thereby includes all relevant information on transport and storage. Relevant information may be number of samples, size of patches, position of the patches, sampling material used, details on impregnation of patches, suppliers etc. Familiarise yourself with the definitions of body and hand exposure as well as potential and actual exposure presented in "A guide to insert and manage content in DermExpoDB".*
field_spl_smpl_mthd_act_body_dtl	**Sampling method actual dermal body exposure - details**	*Provide all relevant details for the method used to sample the specified exposure. This thereby includes all relevant information on transport and storage. Relevant information may be number of samples, size of patches, position of the patches, sampling material used, details on impregnation of patches, suppliers etc. Familiarise yourself with the definitions of body and hand exposure as well as potential and actual exposure presented in "A guide to insert and manage content in DermExpoDB".*
field_spl_ana_mthd_body	**Analytical method dermal body exposure**	*Provide all relevant details of the analytical method used to determine the specified exposure. This thereby includes all relevant information from the point at which the samples are analyzed in the laboratory or on site. Familiarise yourself with the definitions of body and hand exposure presented in "A guide to insert and manage content in DermExpoDB".*
field_spl_unit_ql_body	**Unit QL body**	*Outline the units in which the quantification limits (QL) for the specified sample is inserted in DermExpoDB. Familiarize yourself with the definitions for body and hand exposure outlined in the guide. Predefined categories are available as well as a free text option.*
field_spl_unit_ql_body_other	**Unit QL body - Free text**	
field_spl_ql_head	**Head / face QL**	*Provide the quantification limit(s) (QL) for the specified exposure measurement in the unit specified in the data field „Unit QL body" or „Unit QL body - Free text".*
field_spl_ql_neck	**Neck QL**	*Provide the quantification limit(s) (QL) for the specified exposure measurement in the unit specified in the data field „Unit QL body" or „Unit QL body - Free text".*
field_spl_ql_front	**Front QL**	*Provide the quantification limit(s) (QL) for the specified exposure measurement in the unit specified in the data field „Unit QL body" or „Unit QL body - Free text".*
field_spl_ql_back	**Back QL**	*Provide the quantification limit(s) (QL) for the specified exposure measurement in the unit specified in the data field „Unit QL body" or „Unit QL body - Free text".*
field_spl_ql_arms	**Arms QL**	*Provide the quantification limit(s) (QL) for the specified exposure measurement in the unit specified in the data field „Unit QL body" or „Unit QL body - Free text".*

field_spl_ql_upper_arms	**Upper arms QL**	*Provide the quantification limit(s) (QL) for the specified exposure measurement in the unit specified in the data field „Unit QL body" or „Unit QL body - Free text".*
field_spl_ql_forearms	**Forearms QL**	*Provide the quantification limit(s) (QL) for the specified exposure measurement in the unit specified in the data field „Unit QL body" or „Unit QL body - Free text".*
field_spl_ql_legs	**Legs QL**	*Provide the quantification limit(s) (QL) for the specified exposure measurement in the unit specified in the data field „Unit QL body" or „Unit QL body - Free text".*
field_spl_ql_thighs	**Thighs QL**	*Provide the quantification limit(s) (QL) for the specified exposure measurement in the unit specified in the data field „Unit QL body" or „Unit QL body - Free text".*
field_spl_ql_lower_legs	**Lower legs QL**	*Provide the quantification limit(s) (QL) for the specified exposure measurement in the unit specified in the data field „Unit QL body" or „Unit QL body - Free text".*
field_spl_ql_feet	**Feet QL**	*Provide the quantification limit(s) (QL) for the specified exposure measurement in the unit specified in the data field „Unit QL body" or „Unit QL body - Free text".*
field_spl_smpl_mthd_inhal	**Sampling method inhalation exposure**	*Specify whether personal or stationary sampling was performed. Choose from predefined categories.*
field_spl_smpl_mthd_inhal_dtl	**Sampling method inhalation exposure - details**	*Provide all relevant details for the chosen inhalation sampling method. This thereby includes all relevant information on transport and storage. Other relevant information may be type of sampling material, type of sampling head, flow rate etc.*
field_spl_ana_methd_inhal	**Analytical method inhalation exposure**	*Provide all relevant details of the analytical method used to determine the specified exposure. This thereby includes all relevant information from the point at which the samples are analyzed in the laboratory or on site.*
field_spl_unit_ql_inhal	**Unit QL inhalation exposure**	*Outline the units in which the quantification limits (QL) for the specified sample is inserted in DermExpoDB. Predefined categories are available as well as a free text option.*
field_spl_unit_ql_inhal_other	**Unit QL inhalation exposure - Free text**	
field_spl_inhal_ql	**Inhalation exposure QL**	*Provide the quantification limit(s) (QL) for the specified exposure measurement in the unit specified in the data field „Unit QL body" or „Unit QL body - Free text".*
field_spl_smpl_mthd_surface	**Sampling method surface contamination**	*Provide all relevant details for the chosen surface contamination sampling method. Relevant information may be number of samples, location of samples, surface area of samples, sampling material, pressure applied, number of wipes per sample etc. This thereby includes all relevant information on transport and storage. Enter any relevant quantification limit (QL) (including the unit) in this data field.*

field_spl_ana_mthd_surface	**Analytical method surface contamination**	*Provide all relevant details of the analytical method used to determine the specified exposure. This thereby includes all relevant information from the point at which the samples are analyzed in the laboratory or on site.*
field_spl_smpl_mthd_oral	**Sampling method oral exposure**	*Provide all relevant details for the chosen oral sampling method. This thereby includes all relevant information on transport and storage. Enter any relevant quantification limit (QL) in this data field.*
field_spl_ana_mthd_oral	**Analytical method oral exposure**	*Provide all relevant details of the analytical method used to determine the specified exposure. This thereby includes all relevant information from the point at which the samples are analyzed in the laboratory or on site.*
field_spl_smpl_mthd_biomon	**Sampling method biomonitoring**	*Provide all relevant details for the chosen biomonitoring sampling method. This thereby includes all relevant information on transport and storage. Enter any relevant quantification limit (QL) (including the unit) in this data field.*
field_spl_ana_mthd_biomon	**Analytical method biomonitoring**	*Provide all relevant details of the analytical method used to determine the specified exposure. This thereby includes all relevant information from the point at which the samples are analyzed in the laboratory or on site.*
field_spl_comments	**Other comments on sampling**	*Insert any additional information with regard to sampling and analytics and statistical analysis of data.*
**field_data_owner**	**Dataset owner**	*Specify the institute that owns the data or the institute that has been responsible for extracting published data into the DermExpoDB format.*
**field_data_entry**	**Date of data entry**	*Specify the date on which the data was inserted into DermExpoDB.*
**field_sampling_strategy**	**Sampling strategy**	Indicate whether the sampling was carried out for a specific activity / task / process, shift-based, according to an experimental protocol or according to any other predefined period. In case of pre-shift data, choose „pre-shift“. Choose from predefined categories.
**field_exposure_route**	**Exposure route(s)**	*Indicate whether the measured data is dermal, inhalation or biomonitoring data. For dermal data, distinguish whether data related to hand or body exposure. Familiarize yourself with the definitions for body and hand exposure outlined in "A guide to insert and manage content in DermExpoDB". Choose from predefined categories. It is possible to select more than one element from the predefined list.*
field_sampling_date	**Sampling date**	*Specify the date of sampling in the format DD-MM-JJJJ.*
field_company_nbr	**Company / sampling site number**	*Indicate the unique code for the investigated company / sampling site involved in the measurement preferably as indicated in the reference.*

field_person_nbr	**Person number**	*Indicate the unique code for the worker / volunteer / person involved in the measurement preferably as indicated in the reference.*
field_handedness	**Handedness**	*Indicate the handedness which is an individual's preferential use of one hand also known as the dominant hand.*
field_applied_substance_amount	**Applied amount of substance / agent / ingredient / component**	*Specify the amount / quantity /volume of the chemical substance / agent / ingredient / component (in kg für solids or l for liquids) that was applied during the measurement.*
field_applied_solution_amount	**Applied amount of chemical formulation / application solution / mixture**	*Specify the amount / quantity /volume of the chemical formulation / application solution / mixture (in kg für solids or l for liquids) that was applied during the measurement.*
field_derm_sampling_moment	**Dermal sampling moment**	*Indicate the moment during the (working) day when the sample was collected (e.g. beginning of shift, mid shift, end of shift). Choose from predefined categories.*
field_derm_sampling_time	**Dermal sampling time**	*Provide the duration of sampling for the specified exposure measurement in the unit specified in DermExpoDB.*
field_unit_pde_body	**Unit PDE body**	*Outline the unit in which the measured data for the specifid sample is inserted in DermExpoDB. Familiarize yourself with the definitions for potential and actual exposure as well as hand and body exposure outlined in "A guide to insert and manage content in DermExpoDB". Predefined categories are available as well as a free text option.*
field_unit_pde_body_other	**Unit PDE body - Free text**	
field_head_face_pde	**Head / face PDE**	*Provide the measured amount of the specified exposure for the specified body part and chemical substance / agent / ingredient / component in the unit specified in DermExpoDB. Familiarize yourself with the definitions for potential and actual exposure as well as body and hand exposure outlined in "A guide to insert and manage content in DermExpoDB". If the measured exposure is below the quantification limit (QL), this is stated by inserting "-1". Specify the QL value in the QL data field for the corresponding body part.*

field_neck_pde	**Neck PDE**	*Provide the measured amount of the specified exposure for the specified body part and chemical substance / agent / ingredient / component in the unit specified in DermExpoDB. Familiarize yourself with the definitions for potential and actual exposure as well as body and hand exposure outlined in "A guide to insert and manage content in DermExpoDB". If the measured exposure is below the quantification limit (QL), this is stated by inserting "-1". Specify the QL value in the QL data field for the corresponding body part.*
field_front_pde	**Front torso PDE**	*Provide the measured amount of the specified exposure for the specified body part and chemical substance / agent / ingredient / component in the unit specified in DermExpoDB. Familiarize yourself with the definitions for potential and actual exposure as well as body and hand exposure outlined in "A guide to insert and manage content in DermExpoDB". If the measured exposure is below the quantification limit (QL), this is stated by inserting "-1". Specify the QL value in the QL data field for the corresponding body part.*
field_back_pde	**Back torso PDE**	*Provide the measured amount of the specified exposure for the specified body part and chemical substance / agent / ingredient / component in the unit specified in DermExpoDB. Familiarize yourself with the definitions for potential and actual exposure as well as body and hand exposure outlined in "A guide to insert and manage content in DermExpoDB". If the measured exposure is below the quantification limit (QL), this is stated by inserting "-1". Specify the QL value in the QL data field for the corresponding body part.*
field_left_upper_arm_pde	**Left upper arm PDE**	*Provide the measured amount of the specified exposure for the specified body part and chemical substance / agent / ingredient / component in the unit specified in DermExpoDB. Familiarize yourself with the definitions for potential and actual exposure as well as body and hand exposure outlined in "A guide to insert and manage content in DermExpoDB". If the measured exposure is below the quantification limit (QL), this is stated by inserting "-1". Specify the QL value in the QL data field for the corresponding body part.*
field_right_upper_arm_pde	**Right upper arm PDE**	*Provide the measured amount of the specified exposure for the specified body part and chemical substance / agent / ingredient / component in the unit specified in DermExpoDB. Familiarize yourself with the definitions for potential and actual exposure as well as body and hand exposure outlined in "A guide to insert and manage content in DermExpoDB". If the measured exposure is below the quantification limit (QL), this is stated by inserting "-1". Specify the QL value in the QL data field for the corresponding body part.*

field_upper_arms_pde	**Upper arms PDE**	*Provide the measured amount of the specified exposure for the specified body part and chemical substance / agent / ingredient / component in the unit specified in DermExpoDB. Familiarize yourself with the definitions for potential and actual exposure as well as body and hand exposure outlined in "A guide to insert and manage content in DermExpoDB". If the measured exposure is below the quantification limit (QL), this is stated by inserting "-1". Specify the QL value in the QL data field for the corresponding body part.*
field_left_forearm_pde	**Left forearm PDE**	*Provide the measured amount of the specified exposure for the specified body part and chemical substance / agent / ingredient / component in the unit specified in DermExpoDB. Familiarize yourself with the definitions for potential and actual exposure as well as body and hand exposure outlined in "A guide to insert and manage content in DermExpoDB". If the measured exposure is below the quantification limit (QL), this is stated by inserting "-1". Specify the QL value in the QL data field for the corresponding body part.*
field_right_forearm_pde	**Right forearm PDE**	*Provide the measured amount of the specified exposure for the specified body part and chemical substance / agent / ingredient / component in the unit specified in DermExpoDB. Familiarize yourself with the definitions for potential and actual exposure as well as body and hand exposure outlined in "A guide to insert and manage content in DermExpoDB". If the measured exposure is below the quantification limit (QL), this is stated by inserting "-1". Specify the QL value in the QL data field for the corresponding body part.*
field_forearms_pde	**Forearms PDE**	*Provide the measured amount of the specified exposure for the specified body part and chemical substance / agent / ingredient / component in the unit specified in DermExpoDB. Familiarize yourself with the definitions for potential and actual exposure as well as body and hand exposure outlined in "A guide to insert and manage content in DermExpoDB". If the measured exposure is below the quantification limit (QL), this is stated by inserting "-1". Specify the QL value in the QL data field for the corresponding body part.*
field_left_arm_pde	**Left arm PDE**	*Provide the measured amount of the specified exposure for the specified body part and chemical substance / agent / ingredient / component in the unit specified in DermExpoDB. Familiarize yourself with the definitions for potential and actual exposure as well as body and hand exposure outlined in "A guide to insert and manage content in DermExpoDB". If the measured exposure is below the quantification limit (QL), this is stated by inserting "-1". Specify the QL value in the QL data field for the corresponding body part.*

field_right_arm_pde	**Right arm PDE**	*Provide the measured amount of the specified exposure for the specified body part and chemical substance / agent / ingredient / component in the unit specified in DermExpoDB. Familiarize yourself with the definitions for potential and actual exposure as well as body and hand exposure outlined in "A guide to insert and manage content in DermExpoDB". If the measured exposure is below the quantification limit (QL), this is stated by inserting "-1". Specify the QL value in the QL data field for the corresponding body part.*
field_arms_pde	**Arms PDE**	*Provide the measured amount of the specified exposure for the specified body part and chemical substance / agent / ingredient / component in the unit specified in DermExpoDB. Familiarize yourself with the definitions for potential and actual exposure as well as body and hand exposure outlined in "A guide to insert and manage content in DermExpoDB". If the measured exposure is below the quantification limit (QL), this is stated by inserting "-1". Specify the QL value in the QL data field for the corresponding body part.*
field_left_thigh_pde	**Left thigh PDE**	*Provide the measured amount of the specified exposure for the specified body part and chemical substance / agent / ingredient / component in the unit specified in DermExpoDB. Familiarize yourself with the definitions for potential and actual exposure as well as body and hand exposure outlined in "A guide to insert and manage content in DermExpoDB". If the measured exposure is below the quantification limit (QL), this is stated by inserting "-1". Specify the QL value in the QL data field for the corresponding body part.*
field_right_thigh_pde	**Right thigh PDE**	*Provide the measured amount of the specified exposure for the specified body part and chemical substance / agent / ingredient / component in the unit specified in DermExpoDB. Familiarize yourself with the definitions for potential and actual exposure as well as body and hand exposure outlined in "A guide to insert and manage content in DermExpoDB". If the measured exposure is below the quantification limit (QL), this is stated by inserting "-1". Specify the QL value in the QL data field for the corresponding body part.*
field_thighs_pde	**Thighs PDE**	*Provide the measured amount of the specified exposure for the specified body part and chemical substance / agent / ingredient / component in the unit specified in DermExpoDB. Familiarize yourself with the definitions for potential and actual exposure as well as body and hand exposure outlined in "A guide to insert and manage content in DermExpoDB". If the measured exposure is below the quantification limit (QL), this is stated by inserting "-1". Specify the QL value in the QL data field for the corresponding body part.*

field_left_lower_leg_pde	**Left lower leg PDE**	*Provide the measured amount of the specified exposure for the specified body part and chemical substance / agent / ingredient / component in the unit specified in DermExpoDB. Familiarize yourself with the definitions for potential and actual exposure as well as body and hand exposure outlined in "A guide to insert and manage content in DermExpoDB". If the measured exposure is below the quantification limit (QL), this is stated by inserting "-1". Specify the QL value in the QL data field for the corresponding body part.*
field_right_lower_leg_pde	**Right lower leg PDE**	*Provide the measured amount of the specified exposure for the specified body part and chemical substance / agent / ingredient / component in the unit specified in DermExpoDB. Familiarize yourself with the definitions for potential and actual exposure as well as body and hand exposure outlined in "A guide to insert and manage content in DermExpoDB". If the measured exposure is below the quantification limit (QL), this is stated by inserting "-1". Specify the QL value in the QL data field for the corresponding body part.*
field_lower_legs_pde	**Lower legs PDE**	*Provide the measured amount of the specified exposure for the specified body part and chemical substance / agent / ingredient / component in the unit specified in DermExpoDB. Familiarize yourself with the definitions for potential and actual exposure as well as body and hand exposure outlined in "A guide to insert and manage content in DermExpoDB". If the measured exposure is below the quantification limit (QL), this is stated by inserting "-1". Specify the QL value in the QL data field for the corresponding body part.*
field_left_leg_pde	**Left leg PDE**	*Provide the measured amount of the specified exposure for the specified body part and chemical substance / agent / ingredient / component in the unit specified in DermExpoDB. Familiarize yourself with the definitions for potential and actual exposure as well as body and hand exposure outlined in "A guide to insert and manage content in DermExpoDB". If the measured exposure is below the quantification limit (QL), this is stated by inserting "-1". Specify the QL value in the QL data field for the corresponding body part.*
field_right_leg_pde	**Right leg PDE**	*Provide the measured amount of the specified exposure for the specified body part and chemical substance / agent / ingredient / component in the unit specified in DermExpoDB. Familiarize yourself with the definitions for potential and actual exposure as well as body and hand exposure outlined in "A guide to insert and manage content in DermExpoDB". If the measured exposure is below the quantification limit (QL), this is stated by inserting "-1". Specify the QL value in the QL data field for the corresponding body part.*

field_legs_pde	**Legs PDE**	*Provide the measured amount of the specified exposure for the specified body part and chemical substance / agent / ingredient / component in the unit specified in DermExpoDB. Familiarize yourself with the definitions for potential and actual exposure as well as body and hand exposure outlined in "A guide to insert and manage content in DermExpoDB". If the measured exposure is below the quantification limit (QL), this is stated by inserting "-1". Specify the QL value in the QL data field for the corresponding body part.*
field_feet_pde	**Feet PDE**	*Provide the measured amount of the specified exposure for the specified body part and chemical substance / agent / ingredient / component in the unit specified in DermExpoDB. Familiarize yourself with the definitions for potential and actual exposure as well as body and hand exposure outlined in "A guide to insert and manage content in DermExpoDB". If the measured exposure is below the quantification limit (QL), this is stated by inserting "-1". Specify the QL value in the QL data field for the corresponding body part.*
field_total_body_pde	**Total body PDE**	*Provide the measured amount of the specified exposure for the specified body part and chemical substance / agent / ingredient / component in the unit specified in DermExpoDB. Familiarize yourself with the definitions for potential and actual exposure as well as body and hand exposure outlined in "A guide to insert and manage content in DermExpoDB". If the measured exposure is below the quantification limit (QL), this is stated by inserting "-1". Specify the QL value in the QL data field for the corresponding body part.*
field_number_body_parts_pde	**Number body parts PDE**	*Report the number of potential dermal exposure samples inserted per experiment for the different body parts in DermExpoDB. Familiarize yourself with the definitions for potential and actual exposure as well as body and hand exposure outlined in "A guide to insert and manage content in DermExpoDB".*
field_number_pde_below_loq	**Number body parts PDE below LOQ**	*Report the number of potential dermal exposure samples with values below the limit of quantification (QL) for the given experiment. Familiarize yourself with the definitions for potential and actual exposure as well as body and hand exposure outlined in "A guide to insert and manage content in DermExpoDB".*
field_unit_ade_body	**Unit ADE body**	*Outline the unit in which the measured data for the specifid sample is inserted in DermExpoDB. Familiarize yourself with the definitions for potential and actual exposure as well as hand and body exposure outlined in "A guide to insert and manage content in DermExpoDB". Predefined categories are available as well as a free text option.*

field_unit_ade_body_other	**Unit ADE body - Free text**	
field_head_face_ade	**Head / face ADE**	*Provide the measured amount of the specified exposure for the specified body part and chemical substance / agent / ingredient / component in the unit specified in DermExpoDB. Familiarize yourself with the definitions for potential and actual exposure as well as body and hand exposure outlined in "A guide to insert and manage content in DermExpoDB". If the measured exposure is below the quantification limit (QL), this is stated by inserting "-1". Specify the QL value in the QL data field for the corresponding body part.*
field_neck_ade	**Neck ADE**	*Provide the measured amount of the specified exposure for the specified body part and chemical substance / agent / ingredient / component in the unit specified in DermExpoDB. Familiarize yourself with the definitions for potential and actual exposure as well as body and hand exposure outlined in "A guide to insert and manage content in DermExpoDB". If the measured exposure is below the quantification limit (QL), this is stated by inserting "-1". Specify the QL value in the QL data field for the corresponding body part.*
field_front_ade	**Front torso ADE**	*Provide the measured amount of the specified exposure for the specified body part and chemical substance / agent / ingredient / component in the unit specified in DermExpoDB. Familiarize yourself with the definitions for potential and actual exposure as well as body and hand exposure outlined in "A guide to insert and manage content in DermExpoDB". If the measured exposure is below the quantification limit (QL), this is stated by inserting "-1". Specify the QL value in the QL data field for the corresponding body part.*
field_back_ade	**Back torso ADE**	*Provide the measured amount of the specified exposure for the specified body part and chemical substance / agent / ingredient / component in the unit specified in DermExpoDB. Familiarize yourself with the definitions for potential and actual exposure as well as body and hand exposure outlined in "A guide to insert and manage content in DermExpoDB". If the measured exposure is below the quantification limit (QL), this is stated by inserting "-1". Specify the QL value in the QL data field for the corresponding body part.*

field_left_upper_arm_ade	**Left upper arm ADE**	*Provide the measured amount of the specified exposure for the specified body part and chemical substance / agent / ingredient / component in the unit specified in DermExpoDB. Familiarize yourself with the definitions for potential and actual exposure as well as body and hand exposure outlined in "A guide to insert and manage content in DermExpoDB". If the measured exposure is below the quantification limit (QL), this is stated by inserting "-1". Specify the QL value in the QL data field for the corresponding body part.*
field_right_upper_arm_ade	**Right upper arm ADE**	*Provide the measured amount of the specified exposure for the specified body part and chemical substance / agent / ingredient / component in the unit specified in DermExpoDB. Familiarize yourself with the definitions for potential and actual exposure as well as body and hand exposure outlined in "A guide to insert and manage content in DermExpoDB". If the measured exposure is below the quantification limit (QL), this is stated by inserting "-1". Specify the QL value in the QL data field for the corresponding body part.*
field_upper_arms_ade	**Upper arms ADE**	*Provide the measured amount of the specified exposure for the specified body part and chemical substance / agent / ingredient / component in the unit specified in DermExpoDB. Familiarize yourself with the definitions for potential and actual exposure as well as body and hand exposure outlined in "A guide to insert and manage content in DermExpoDB". If the measured exposure is below the quantification limit (QL), this is stated by inserting "-1". Specify the QL value in the QL data field for the corresponding body part.*
field_left_forearm_ade	**Left forearm ADE**	*Provide the measured amount of the specified exposure for the specified body part and chemical substance / agent / ingredient / component in the unit specified in DermExpoDB. Familiarize yourself with the definitions for potential and actual exposure as well as body and hand exposure outlined in "A guide to insert and manage content in DermExpoDB". If the measured exposure is below the quantification limit (QL), this is stated by inserting "-1". Specify the QL value in the QL data field for the corresponding body part.*
field_right_forearm_ade	**Right forearm ADE**	*Provide the measured amount of the specified exposure for the specified body part and chemical substance / agent / ingredient / component in the unit specified in DermExpoDB. Familiarize yourself with the definitions for potential and actual exposure as well as body and hand exposure outlined in "A guide to insert and manage content in DermExpoDB". If the measured exposure is below the quantification limit (QL), this is stated by inserting "-1". Specify the QL value in the QL data field for the corresponding body part.*

field_forearms_ade	**Forearms ADE**	*Provide the measured amount of the specified exposure for the specified body part and chemical substance / agent / ingredient / component in the unit specified in DermExpoDB. Familiarize yourself with the definitions for potential and actual exposure as well as body and hand exposure outlined in "A guide to insert and manage content in DermExpoDB". If the measured exposure is below the quantification limit (QL), this is stated by inserting "-1". Specify the QL value in the QL data field for the corresponding body part.*
field_left_arm_ade	**Left arm ADE**	*Provide the measured amount of the specified exposure for the specified body part and chemical substance / agent / ingredient / component in the unit specified in DermExpoDB. Familiarize yourself with the definitions for potential and actual exposure as well as body and hand exposure outlined in "A guide to insert and manage content in DermExpoDB". If the measured exposure is below the quantification limit (QL), this is stated by inserting "-1". Specify the QL value in the QL data field for the corresponding body part.*
field_right_arm_ade	**Right arm ADE**	*Provide the measured amount of the specified exposure for the specified body part and chemical substance / agent / ingredient / component in the unit specified in DermExpoDB. Familiarize yourself with the definitions for potential and actual exposure as well as body and hand exposure outlined in "A guide to insert and manage content in DermExpoDB". If the measured exposure is below the quantification limit (QL), this is stated by inserting "-1". Specify the QL value in the QL data field for the corresponding body part.*
field_arms_ade	**Arms ADE**	*Provide the measured amount of the specified exposure for the specified body part and chemical substance / agent / ingredient / component in the unit specified in DermExpoDB. Familiarize yourself with the definitions for potential and actual exposure as well as body and hand exposure outlined in "A guide to insert and manage content in DermExpoDB". If the measured exposure is below the quantification limit (QL), this is stated by inserting "-1". Specify the QL value in the QL data field for the corresponding body part.*
field_left_thigh_ade	**Left thigh ADE**	*Provide the measured amount of the specified exposure for the specified body part and chemical substance / agent / ingredient / component in the unit specified in DermExpoDB. Familiarize yourself with the definitions for potential and actual exposure as well as body and hand exposure outlined in "A guide to insert and manage content in DermExpoDB". If the measured exposure is below the quantification limit (QL), this is stated by inserting "-1". Specify the QL value in the QL data field for the corresponding body part.*

field_right_thigh_ade	**Right thigh ADE**	*Provide the measured amount of the specified exposure for the specified body part and chemical substance / agent / ingredient / component in the unit specified in DermExpoDB. Familiarize yourself with the definitions for potential and actual exposure as well as body and hand exposure outlined in "A guide to insert and manage content in DermExpoDB". If the measured exposure is below the quantification limit (QL), this is stated by inserting "-1". Specify the QL value in the QL data field for the corresponding body part.*
field_thighs_ade	**Thighs ADE**	*Provide the measured amount of the specified exposure for the specified body part and chemical substance / agent / ingredient / component in the unit specified in DermExpoDB. Familiarize yourself with the definitions for potential and actual exposure as well as body and hand exposure outlined in "A guide to insert and manage content in DermExpoDB". If the measured exposure is below the quantification limit (QL), this is stated by inserting "-1". Specify the QL value in the QL data field for the corresponding body part.*
field_left_lower_leg_ade	**Left lower leg ADE**	*Provide the measured amount of the specified exposure for the specified body part and chemical substance / agent / ingredient / component in the unit specified in DermExpoDB. Familiarize yourself with the definitions for potential and actual exposure as well as body and hand exposure outlined in "A guide to insert and manage content in DermExpoDB". If the measured exposure is below the quantification limit (QL), this is stated by inserting "-1". Specify the QL value in the QL data field for the corresponding body part.*
field_right_lower_leg_ade	**Right lower leg ADE**	*Provide the measured amount of the specified exposure for the specified body part and chemical substance / agent / ingredient / component in the unit specified in DermExpoDB. Familiarize yourself with the definitions for potential and actual exposure as well as body and hand exposure outlined in "A guide to insert and manage content in DermExpoDB". If the measured exposure is below the quantification limit (QL), this is stated by inserting "-1". Specify the QL value in the QL data field for the corresponding body part.*
field_lower_legs_ade	**Lower legs ADE**	*Provide the measured amount of the specified exposure for the specified body part and chemical substance / agent / ingredient / component in the unit specified in DermExpoDB. Familiarize yourself with the definitions for potential and actual exposure as well as body and hand exposure outlined in "A guide to insert and manage content in DermExpoDB". If the measured exposure is below the quantification limit (QL), this is stated by inserting "-1". Specify the QL value in the QL data field for the corresponding body part.*

field_left_leg_ade	**Left leg ADE**	*Provide the measured amount of the specified exposure for the specified body part and chemical substance / agent / ingredient / component in the unit specified in DermExpoDB. Familiarize yourself with the definitions for potential and actual exposure as well as body and hand exposure outlined in "A guide to insert and manage content in DermExpoDB". If the measured exposure is below the quantification limit (QL), this is stated by inserting "-1". Specify the QL value in the QL data field for the corresponding body part.*
field_right_leg_ade	**Right leg ADE**	*Provide the measured amount of the specified exposure for the specified body part and chemical substance / agent / ingredient / component in the unit specified in DermExpoDB. Familiarize yourself with the definitions for potential and actual exposure as well as body and hand exposure outlined in "A guide to insert and manage content in DermExpoDB". If the measured exposure is below the quantification limit (QL), this is stated by inserting "-1". Specify the QL value in the QL data field for the corresponding body part.*
field_legs_ade	**Legs ADE**	*Provide the measured amount of the specified exposure for the specified body part and chemical substance / agent / ingredient / component in the unit specified in DermExpoDB. Familiarize yourself with the definitions for potential and actual exposure as well as body and hand exposure outlined in "A guide to insert and manage content in DermExpoDB". If the measured exposure is below the quantification limit (QL), this is stated by inserting "-1". Specify the QL value in the QL data field for the corresponding body part.*
field_feet_ade	**Feet ADE**	*Provide the measured amount of the specified exposure for the specified body part and chemical substance / agent / ingredient / component in the unit specified in DermExpoDB. Familiarize yourself with the definitions for potential and actual exposure as well as body and hand exposure outlined in "A guide to insert and manage content in DermExpoDB". If the measured exposure is below the quantification limit (QL), this is stated by inserting "-1". Specify the QL value in the QL data field for the corresponding body part.*
field_total_body_ade	**Total body ADE**	*Provide the measured amount of the specified exposure for the specified body part and chemical substance / agent / ingredient / component in the unit specified in DermExpoDB. Familiarize yourself with the definitions for potential and actual exposure as well as body and hand exposure outlined in "A guide to insert and manage content in DermExpoDB". If the measured exposure is below the quantification limit (QL), this is stated by inserting "-1". Specify the QL value in the QL data field for the corresponding body part.*

field_number_body_parts_ade	**Number body parts ADE**	*Report the number of actual dermal exposure samples inserted per experiment for the different body parts in DermExpoDB. Familiarize yourself with the definitions for potential and actual exposure as well as body and hand exposure outlined in "A guide to insert and manage content in DermExpoDB".*
field_number_ade_below_loq	**Number body parts ADE below LOQ**	*Report the number of actual dermal exposure samples with values below the limit of quantification (QL) for the given experiment. Familiarize yourself with the definitions for potential and actual exposure as well as body and hand exposure outlined in "A guide to insert and manage content in DermExpoDB".*
field_unit_pde_hands	**Unit PDE hands**	*Outline the unit in which the measured data for the specifid sample is inserted in DermExpoDB. Familiarize yourself with the definitions for potential and actual exposure as well as hand and body exposure outlined in "A guide to insert and manage content in DermExpoDB". Predefined categories are available as well as a free text option.*
field_unit_pde_hands_other	**Unit PDE hands - Free text**	
field_hands_pde	**Hands PDE**	*Provide the measured amount of the specified exposure for the specified body part and chemical substance / agent / ingredient / component in the unit specified in DermExpoDB. Familiarize yourself with the definitions for potential and actual exposure as well as body and hand exposure outlined in "A guide to insert and manage content in DermExpoDB". If the measured exposure is below the quantification limit (QL), this is stated by inserting "-1". Specify the QL value in the QL data field for the corresponding body part.*
field_left_hand_pde	**Left hand PDE**	*Provide the measured amount of the specified exposure for the specified body part and chemical substance / agent / ingredient / component in the unit specified in DermExpoDB. Familiarize yourself with the definitions for potential and actual exposure as well as body and hand exposure outlined in "A guide to insert and manage content in DermExpoDB". If the measured exposure is below the quantification limit (QL), this is stated by inserting "-1". Specify the QL value in the QL data field for the corresponding body part.*
field_right_hand_pde	**Right hand PDE**	*Provide the measured amount of the specified exposure for the specified body part and chemical substance / agent / ingredient / component in the unit specified in DermExpoDB. Familiarize yourself with the definitions for potential and actual exposure as well as body and hand exposure outlined in "A guide to insert and manage content in DermExpoDB". If the measured exposure is below the quantification limit (QL), this is stated by inserting "-1". Specify the QL value in the QL data field for the corresponding body part.*

field_unit_ade_hands	**Unit ADE hands**	*Outline the unit in which the measured data for the specifid sample is inserted in DermExpoDB. Familiarize yourself with the definitions for potential and actual exposure as well as hand and body exposure outlined in "A guide to insert and manage content in DermExpoDB". Predefined categories are available as well as a free text option.*
field_unit_ade_hands_other	**Unit ADE hands - Free text**	
field_hands_ade	**Hands ADE**	*Provide the measured amount of the specified exposure for the specified body part and chemical substance / agent / ingredient / component in the unit specified in DermExpoDB. Familiarize yourself with the definitions for potential and actual exposure as well as body and hand exposure outlined in "A guide to insert and manage content in DermExpoDB". If the measured exposure is below the quantification limit (QL), this is stated by inserting "-1". Specify the QL value in the QL data field for the corresponding body part.*
field_left_hand_ade	**Left hand ADE**	*Provide the measured amount of the specified exposure for the specified body part and chemical substance / agent / ingredient / component in the unit specified in DermExpoDB. Familiarize yourself with the definitions for potential and actual exposure as well as body and hand exposure outlined in "A guide to insert and manage content in DermExpoDB". If the measured exposure is below the quantification limit (QL), this is stated by inserting "-1". Specify the QL value in the QL data field for the corresponding body part.*
field_right_hand_ade	**Right hand ADE**	*Provide the measured amount of the specified exposure for the specified body part and chemical substance / agent / ingredient / component in the unit specified in DermExpoDB. Familiarize yourself with the definitions for potential and actual exposure as well as body and hand exposure outlined in "A guide to insert and manage content in DermExpoDB". If the measured exposure is below the quantification limit (QL), this is stated by inserting "-1". Specify the QL value in the QL data field for the corresponding body part.*

field_head_face_sa	**Head / face SA**	*Specify the surface area of the specified sampler in case a surrogate sampling techniques was applied or the sampled body part in case a removal sampling techniques was used. If the sampling was carried out with a surrogate sampling technique (e.g. overalls or gloves), the measured data relates to the surface area of the clothing area and thus the area of the sampler (e.g. area of the overall measured by weighing) must therefore be specified. If a removal method (e.g. tape stripping or hand washing) has been performed, the measured data relate to the specified body surface area, which then has to be reported. Enter only data in DermExpoDB that is related to the specified sampler surface (e.g. µg per sampler or sampled surface or µg divided by sampler or sampled surface area). Avoid inserting data into DermExpoDB that has been extrapolated in some other way, e.g. measured value determined with a coverall divided by a standardized body surface area.*
field_neck_sa	**Neck SA**	*Specify the surface area of the specified sampler in case a surrogate sampling techniques was applied or the sampled body part in case a removal sampling techniques was used. If the sampling was carried out with a surrogate sampling technique (e.g. overalls or gloves), the measured data relates to the surface area of the clothing area and thus the area of the sampler (e.g. area of the overall measured by weighing) must therefore be specified. If a removal method (e.g. tape stripping or hand washing) has been performed, the measured data relate to the specified body surface area, which then has to be reported. Enter only data in DermExpoDB that is related to the specified sampler surface (e.g. µg per sampler or sampled surface or µg divided by sampler or sampled surface area). Avoid inserting data into DermExpoDB that has been extrapolated in some other way, e.g. measured value determined with a coverall divided by a standardized body surface area.*

field_front_sa	**Front torso SA**	*Specify the surface area of the specified sampler in case a surrogate sampling techniques was applied or the sampled body part in case a removal sampling techniques was used.<br>If the sampling was carried out with a surrogate sampling technique (e.g. overalls or gloves), the measured data relates to the surface area of the clothing area and thus the area of the sampler (e.g. area of the overall measured by weighing) must therefore be specified. If a removal method (e.g. tape stripping or hand washing) has been performed, the measured data relate to the specified body surface area, which then has to be reported. Enter only data in DermExpoDB that is related to the specified sampler surface (e.g. µg per sampler or sampled surface or µg divided by sampler or sampled surface area). Avoid inserting data into DermExpoDB that has been extrapolated in some other way, e.g. measured value determined with a coverall divided by a standardized body surface area.*
field_back_sa	**Back torso SA**	*Specify the surface area of the specified sampler in case a surrogate sampling techniques was applied or the sampled body part in case a removal sampling techniques was used.<br>If the sampling was carried out with a surrogate sampling technique (e.g. overalls or gloves), the measured data relates to the surface area of the clothing area and thus the area of the sampler (e.g. area of the overall measured by weighing) must therefore be specified. If a removal method (e.g. tape stripping or hand washing) has been performed, the measured data relate to the specified body surface area, which then has to be reported. Enter only data in DermExpoDB that is related to the specified sampler surface (e.g. µg per sampler or sampled surface or µg divided by sampler or sampled surface area). Avoid inserting data into DermExpoDB that has been extrapolated in some other way, e.g. measured value determined with a coverall divided by a standardized body surface area.*

field_upper_arms_sa	**Upper arms SA**	*Specify the surface area of the specified sampler in case a surrogate sampling techniques was applied or the sampled body part in case a removal sampling techniques was used.<br>If the sampling was carried out with a surrogate sampling technique (e.g. overalls or gloves), the measured data relates to the surface area of the clothing area and thus the area of the sampler (e.g. area of the overall measured by weighing) must therefore be specified. If a removal method (e.g. tape stripping or hand washing) has been performed, the measured data relate to the specified body surface area, which then has to be reported. Enter only data in DermExpoDB that is related to the specified sampler surface (e.g. µg per sampler or sampled surface or µg divided by sampler or sampled surface area). Avoid inserting data into DermExpoDB that has been extrapolated in some other way, e.g. measured value determined with a coverall divided by a standardized body surface area.*
field_forearms_sa	**Forearms SA**	*Specify the surface area of the specified sampler in case a surrogate sampling techniques was applied or the sampled body part in case a removal sampling techniques was used.<br>If the sampling was carried out with a surrogate sampling technique (e.g. overalls or gloves), the measured data relates to the surface area of the clothing area and thus the area of the sampler (e.g. area of the overall measured by weighing) must therefore be specified. If a removal method (e.g. tape stripping or hand washing) has been performed, the measured data relate to the specified body surface area, which then has to be reported. Enter only data in DermExpoDB that is related to the specified sampler surface (e.g. µg per sampler or sampled surface or µg divided by sampler or sampled surface area). Avoid inserting data into DermExpoDB that has been extrapolated in some other way, e.g. measured value determined with a coverall divided by a standardized body surface area.*

field_arms_sa	**Arms SA**	*Specify the surface area of the specified sampler in case a surrogate sampling techniques was applied or the sampled body part in case a removal sampling techniques was used.<br>If the sampling was carried out with a surrogate sampling technique (e.g. overalls or gloves), the measured data relates to the surface area of the clothing area and thus the area of the sampler (e.g. area of the overall measured by weighing) must therefore be specified. If a removal method (e.g. tape stripping or hand washing) has been performed, the measured data relate to the specified body surface area, which then has to be reported. Enter only data in DermExpoDB that is related to the specified sampler surface (e.g. µg per sampler or sampled surface or µg divided by sampler or sampled surface area). Avoid inserting data into DermExpoDB that has been extrapolated in some other way, e.g. measured value determined with a coverall divided by a standardized body surface area.*
field_thighs_sa	**Thighs SA**	*Specify the surface area of the specified sampler in case a surrogate sampling techniques was applied or the sampled body part in case a removal sampling techniques was used.<br>If the sampling was carried out with a surrogate sampling technique (e.g. overalls or gloves), the measured data relates to the surface area of the clothing area and thus the area of the sampler (e.g. area of the overall measured by weighing) must therefore be specified. If a removal method (e.g. tape stripping or hand washing) has been performed, the measured data relate to the specified body surface area, which then has to be reported. Enter only data in DermExpoDB that is related to the specified sampler surface (e.g. µg per sampler or sampled surface or µg divided by sampler or sampled surface area). Avoid inserting data into DermExpoDB that has been extrapolated in some other way, e.g. measured value determined with a coverall divided by a standardized body surface area.*

field_lower_legs_sa	**Lower legs SA**	*Specify the surface area of the specified sampler in case a surrogate sampling techniques was applied or the sampled body part in case a removal sampling techniques was used.<br>If the sampling was carried out with a surrogate sampling technique (e.g. overalls or gloves), the measured data relates to the surface area of the clothing area and thus the area of the sampler (e.g. area of the overall measured by weighing) must therefore be specified. If a removal method (e.g. tape stripping or hand washing) has been performed, the measured data relate to the specified body surface area, which then has to be reported. Enter only data in DermExpoDB that is related to the specified sampler surface (e.g. µg per sampler or sampled surface or µg divided by sampler or sampled surface area). Avoid inserting data into DermExpoDB that has been extrapolated in some other way, e.g. measured value determined with a coverall divided by a standardized body surface area.*
field_legs_sa	**Legs SA**	*Specify the surface area of the specified sampler in case a surrogate sampling techniques was applied or the sampled body part in case a removal sampling techniques was used.<br>If the sampling was carried out with a surrogate sampling technique (e.g. overalls or gloves), the measured data relates to the surface area of the clothing area and thus the area of the sampler (e.g. area of the overall measured by weighing) must therefore be specified. If a removal method (e.g. tape stripping or hand washing) has been performed, the measured data relate to the specified body surface area, which then has to be reported. Enter only data in DermExpoDB that is related to the specified sampler surface (e.g. µg per sampler or sampled surface or µg divided by sampler or sampled surface area). Avoid inserting data into DermExpoDB that has been extrapolated in some other way, e.g. measured value determined with a coverall divided by a standardized body surface area.*

field_feet_sa	**Feet SA**	*Specify the surface area of the specified sampler in case a surrogate sampling techniques was applied or the sampled body part in case a removal sampling techniques was used.<br>If the sampling was carried out with a surrogate sampling technique (e.g. overalls or gloves), the measured data relates to the surface area of the clothing area and thus the area of the sampler (e.g. area of the overall measured by weighing) must therefore be specified. If a removal method (e.g. tape stripping or hand washing) has been performed, the measured data relate to the specified body surface area, which then has to be reported. Enter only data in DermExpoDB that is related to the specified sampler surface (e.g. µg per sampler or sampled surface or µg divided by sampler or sampled surface area). Avoid inserting data into DermExpoDB that has been extrapolated in some other way, e.g. measured value determined with a coverall divided by a standardized body surface area.*
field_total_body_sa	**Total body SA**	*Specify the surface area of the specified sampler in case a surrogate sampling techniques was applied or the sampled body part in case a removal sampling techniques was used.<br>If the sampling was carried out with a surrogate sampling technique (e.g. overalls or gloves), the measured data relates to the surface area of the clothing area and thus the area of the sampler (e.g. area of the overall measured by weighing) must therefore be specified. If a removal method (e.g. tape stripping or hand washing) has been performed, the measured data relate to the specified body surface area, which then has to be reported. Enter only data in DermExpoDB that is related to the specified sampler surface (e.g. µg per sampler or sampled surface or µg divided by sampler or sampled surface area). Avoid inserting data into DermExpoDB that has been extrapolated in some other way, e.g. measured value determined with a coverall divided by a standardized body surface area.*

field_hands_sa	**Hands SA**	*Specify the surface area of the specified sampler in case a surrogate sampling techniques was applied or the sampled body part in case a removal sampling techniques was used.*<br>*If the sampling was carried out with a surrogate sampling technique (e.g. overalls or gloves), the measured data relates to the surface area of the clothing area and thus the area of the sampler (e.g. area of the overall measured by weighing) must therefore be specified. If a removal method (e.g. tape stripping or hand washing) has been performed, the measured data relate to the specified body surface area, which then has to be reported. Enter only data in DermExpoDB that is related to the specified sampler surface (e.g. µg per sampler or sampled surface or µg divided by sampler or sampled surface area). Avoid inserting data into DermExpoDB that has been extrapolated in some other way, e.g. measured value determined with a coverall divided by a standardized body surface area.*
field_inhal_sampling_time	**Inhalation sampling time**	*Provide the duration of sampling for the specified exposure measurement in the unit specified in DermExpoDB.*
field_unit_inhal	**Unit Inhalation exposure**	*Outline the unit in which the measured data for the specifid sample is inserted in DermExpoDB. Predefined categories are available as well as a free text option.*
field_unit_inhal_other	**Unit Inhalation exposure - Free text**	
field_inhalation_expo_level	**Inhalation exposure**	*Provide the measured inhalation exposure in the unit specified in DermExpoDB. When not stated otherwise, the given exposure relates to the chemical substance / agent / ingredient / component. For aerosol exposure, use data fields "inhalable exposure" and "respirable exposure".*
field_inhalable_expo_level	**Inhalable exposure**	*Provide the measured inhalable aerosol exposure in the unit specified in DermExpoDB. When not stated otherwise, the given exposure relates to the chemical substance / agent / ingredient / component.*
field_respirable_expo_level	**Respirable exposure**	*Provide the measured respirable aerosol exposure in the unit specified in DermExpoDB. When not stated otherwise, the given exposure relates to the chemical substance / agent / ingredient / component.*
field_biomon_sampling_time	**Biomonitoring sampling time**	*Provide the duration of sampling for the specified exposure measurement in the unit specified in DermExpoDB.*
field_unit_biomon	**Unit biomonitoring exposure**	*Outline the unit in which the measured data for the specifid sample is inserted in DermExpoDB.*

field_biomon_expo_level	**Biomonitoring exposure**	*Provide the measured exposure by biomonitoring in the unit specified in DermExpoDB. When not stated otherwise, the given exposure relates to the chemical substance / agent / ingredient / component.*
field_unit_oral_expo	**Unit oral exposure**	*Outline the unit in which the measured data for the specifid sample is inserted in DermExpoDB.*
field_oral_expo_level	**Oral exposure**	*Provide the measured oral exposure in the unit specified in DermExpoDB. When not stated otherwise, the given exposure relates to the chemical substance / agent / ingredient / component.*
field_unit_surface_contam	**Unit surface contamination**	*Outline the unit in which the measured data for the specifid sample is inserted in DermExpoDB.*
field_surface_contam_level	**Surface contamination**	*Provide the measured surface contamination in the unit specified in DermExpoDB. When not stated otherwise, the given exposure relates to the chemical substance / agent / ingredient / component.*
field_raw_comments	**Other comments on raw data**	*Address any other information that may be relevant and related to the measured data, e.g. when measured values were adjusted in some way.*

Mandatory fields are marked in bold in the 'Machine Name' column.

Supplementary Material 5 – ER diagram for summary data

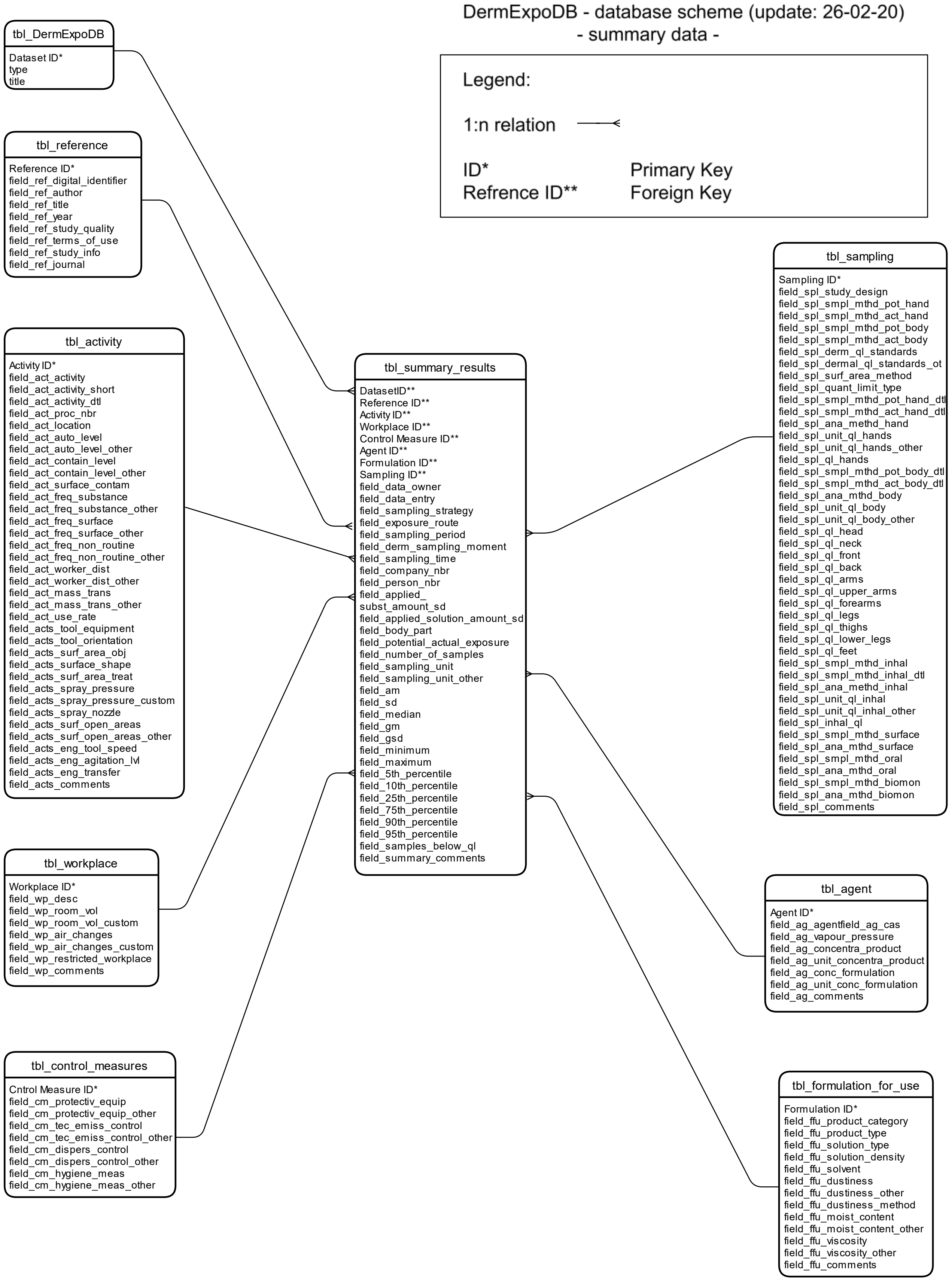

Supplementary Material 6 – data field list for summary data

Machine name	Field name	Field input description
**type**	**Type of data**	*Specify the type of data that is uploaded in DermExpoDB.*
**title**	**Dataset title**	*Specify a unique title for each data record.*
**field_ref_digital_identifier**	**Digital identifier**	*Include DOI or ISBN or web address if the data is publically available. If it is not publicly available, insert "unpublished".*
**field_ref_author**	**Author(s) of study**	*Name the author(s) which originally published the data. Preferred format is: Fritz DH, Mayer JJ.*
**field_ref_title**	**Reference title**	*Provide the title of the report/publication in which the data was published.*
**field_ref_year**	**Year**	*Specify the year in which study or data was published.*
**field_ref_study_quality**	**Study quality**	*Judge the quality of the study.*
**field_ref_terms_of_use**	**Terms of use of data**	*Decide whether your data is flagged as confidential or non-confidential data upon upload in the database software application. Outside parties can only request access to non-confidential data. Data indicated as being confidential upon upload may not be used without the approval by the Steering Committee by any user.*
field_ref_study_info	**Abstract / background information of study**	*Submit an abstract or executive summary of the study.*
field_ref_journal	**Journal / Report**	*Identify the journal or publisher that published the data. Preferred format is journal name, volume (issue): pages or publisher identifier.*
**field_act_activity**	**Activity**	*Provide the activity category. It is possible to select more than one element from the predefined list.*
**field_act_activity_short**	**Activity - Short description**	*Provide a brief description of the activity performed during the measurement.*
**field_act_activity_dtl**	**Activity - details**	*Give a detailed description including all relevant contextual information of the activity, work tasks or processes performed during the measurement.*
**field_act_proc_nbr**	**PROC number & PROC description**	*Select the PROC number / PROC description that is relevant for the major activity, i.e. which dominates the exposure. It is possible to select more than one element from the predefined list.*
**field_act_location**	**Location**	*Distinguish whether an indoor or outdoor application or both was carried out.*
field_act_auto_level	**Level of automation**	*Record the degree of automation of the process. Predefined categories are available as well as a free text option.*

field_act_auto_level_other	**Level of automation - Free text**	
field_act_contain_level	**Level of containment**	*Record the degree of enclosure of an activity or process. Predefined categories are available as well as a free text option.*
field_act_contain_level_other	**Level of containment - Free text**	
field_act_surface_contam	**Surface contamination**	*Describe visible, measured or judged surface contamination (contaminated surfaces) with which the worker comes into contact during the activity. Relevent information are i.e. level of contamination or % surface area contaminated. Reference to available quantitative, measured surface contamination data.*
field_act_freq_substance	**Contact frequency with chemical substance / product / formulation**	*Provide recorded information or own judgement on frequency of routine contact with the chemical product / formulation / application solution / mixture / substance / agent / ingredient / component e.g. direct contact by touching solid objects or immersion of body parts in chemical. Predefined categories are available as well as a free text option.*
field_act_freq_substance_other	**Contact frequency with chemical substance / product / formulation - Free text**	
field_act_freq_surface	**Contact frequency with contaminated surfaces**	*Provide recorded information or own judgement on frequency of routine contact with equipment, tools, control panels or or other contaminated objects. Predefined categories are available as well as a free text option.*
field_act_freq_surface_other	**Contact frequency with contaminated surfaces - Free text**	
field_act_freq_non_routine	**Contact frequency due to non-routine activities**	*Provide recorded information or own judgement on frequency of non-routine contact such as contact during trouble shooting, maintenance or repairs (e.g. unblocking nozzles). Predefined categories are available as well as a free text option.*
field_act_freq_non_routine_other	**Contact frequency due to non-routine activities - Free text**	

field_act_worker_dist	**Distance worker-source**	*Estimate the distance between worker and emission source. Predefined categories are available as well as a free text option.*
field_act_worker_dist_other	**Distance worker-source - Free text**	
field_act_mass_trans	**Relevant mass transport processes**	*Specify the mass transport processes that were observed during the measurement. For description of dermal mass transport processes: Schneider T, Vermeulen R, Brouwer DH, Cherrie JW, Kromhout H, Fogh CL. Conceptual model for assessment of dermal exposure. Occup Environ Med. 1999 Nov;56(11):765-73. doi: 10.1136/oem.56.11.765. PMID: 10658563; PMCID: PMC1757678. Predefined categories are available as well as a free text option. It is possible to select more than one element from the predefined list.*
field_act_mass_trans_other	**Relevant mass transport processes - Free text**	
field_act_use_rate	**Product use rate / scale**	*Specify the (recommended) use rate for the handled product (preferably in kg/min for solids or L/min for liquids). Please also include the unit.*
field_acts_tool_equipment	**Activities with tools: Equipment**	*Only relevant for activities where tools are used: Give a detailed description of all equipment that was used during the measurement, i.e. spray equipment or long-handle brush.*
field_acts_tool_orientation	**Activities with tools: Orientation of work**	*Only relevant for activities where tools are used: Indicate the direction or orientation in which a product is applied in relation to worker. Choose from predefined categories.*
field_acts_surf_area_obj	**Activities with treated objects: Area of treated objects**	*Only relevant for activities where objects are treated: Specify the surface area of the object (in m^2) that was treated during the measurement.*
field_acts_surface_shape	**Activities with treated objects: Surface shape of treated objects**	*Only relevant for activities where objects are treated: Outline the shape of the object treated e.g. by spraying. Choose from predefined categories.*
field_acts_surf_area_treat	**Activities with treated surfaces: Area of treated (flat) surface**	*Only relevant for activities where (flat) surface areas are treated: Specify the area of the (flat) surface (in m^2) that was treated during the measurement.*

field_acts_spray_pressure	**Spraying: Spray pressure**	*Only relevant for spray activities: Specify the spray pressure (in bar) that was set during the measurement on the spraying device. Predefined categories are available as well as a numerical input option.*
field_acts_spray_pressure_custom	**Spraying: Spray pressure - Numerical value**	
field_acts_spray_nozzle	**Spraying: Spray nozzle**	*Only relevant for spray activities: Give information on the spray nozzle (i.e. name of spray nozzle, spray nozzle area, spray nozzle angle) attached to the spraying device that was used during the measurement.*
field_acts_surf_open_areas	**Activities with open surfaces: Area of open chemical substance / product / formulation**	*Only relevant for activities with open chemical substance / agent / ingredient / component / product / formulation / application solution / mixture surface: Enter the area of the open surface area (in m^2), for example if the chemical substance / agent / ingredient/ component / product / formulation / application solution / mixture is stored in an open container, jar or vessel. Predefined categories are available as well as a numerical input option.*
field_acts_surf_open_areas_other	**Activities with open surfaces: Area of open chemical substance / product / formulation - Numerical value**	
field_acts_eng_tool_speed	**Activities with energy input: Tool speed**	*Only relevant for activities with energy input: Specify the (rotational) speed of the tool used during the measurement which can lead to an increased release of a chemical substance / agent / ingredient / component from a tool (such as spattering from rollers).*
field_acts_eng_agitation_lvl	**Activities with energy input: Agitation level**	*Only relevant for activities with energy input: Provide the level of agitation of a liquid during an activity (e.g. mixing). Choose from predefined categories.*
field_acts_eng_transfer	**Activities with energy input: Transfer type**	*Only relevant for transfer of liquids and solids: Outline the type of technique applied to transfer liquids or solids. Choose from predefined categories.*
field_acts_comments	**Other comments on activity**	*Address any other information that may be relevant and related to the activities performed during the measurement.*
**field_wp_desc**	**Workplace description**	*Provide a comprehensive description of the workplace where the measurement took place.*

field_wp_room_vol	**Indoor room volume**	*If applicable, specify the indoor air volume. Predefined categories are available as well as a numerical input option.*
field_wp_room_vol_custom	**Indoor room volume - Numerical value**	
field_wp_air_changes	**Indoor air changes / outdoors wind speed**	*For indoor applications, provide the ventilation rate in the room in which the measurement was performed. For outdoor applications, provide the wind speed. Predefined categories are available as well as a numerical input option.*
field_wp_air_changes_custom	**Indoor air changes / outdoors wind speed - Numerical value**	
field_wp_restricted_workplace	**Restricted workplace**	*Indicate whether work was performed in a restricted workspace with an increased risk of skin contact, e.g. low hanging crop, spaces entered on the knees or similar. Choose from predefined categories.*
field_wp_comments	**Other comments on workplace**	*Address any other information that may be relevant and related to the workplace where the measurement was performed.*
**field_cm_protectiv_equip**	**Personal Protective Equipment**	*Provide information on the personal protective equipment (PPE) used during the measurement. It is possible to select more than one element from the predefined list.*
field_cm_protectiv_equip_other	**Personal Protective Equipment - Free text**	
field_cm_tec_emiss_control	**Technical emission control at source**	*Describe any technical control measure at the source that was present during the measurement. Predefined categories are available as well as a free text option. It is possible to select more than one element from the predefined list.*
field_cm_tec_emiss_control_other	**Technical emission control at source - Free text**	
field_cm_dispers_control	**Dispersion control away from source**	*Describe any controls related to controlling the dispersion of air contaminants in the work environment, e.g. improved general ventilation that was present during the measurement. Predefined categories are available as well as a free text option. It is possible to select more than one element from the predefined list.*

field_cm_dispers_control_other	**Dispersion control away from source – Free text**	
field_cm_hygiene_meas	**Hygiene measures**	*Describe any hygiene measures that that have been implemented in the workplace. Predefined categories are available as well as a free text option. It is possible to select more than one element from the predefined list.*
field_cm_hygiene_meas_other	**Hygiene measures - free text**	
**field_ag_agent**	**Chemical substance / agent / ingredient / component**	*Indicate the name of the chemical substance / agent / ingredient / component to which the measured exposure refers. If more than one chemical substance / agent / ingredient / component has been measured for a given exposure situation, indicate only the chemical substance / agent / ingredient / component to which the exposure value of the data record refers to. If the measured exposure refers to a chemical product / formulation / application solution / mixture, e.g. heavy fuel oils (HFO), and napthalene was measured as an indicator, indicate accordingly, e.g. "heavy fuel oils (HFO) (napthalene as marker for exposure to HFO)".*
**field_ag_cas**	**CAS**	*Provide the CAS (Chemical Abstracts Service) number of the chemical of interest. In case a CAS is not assignable, e.g. because the measured exposure relates to a chemical product, insert „not assignable“.*
**field_ag_vapour_pressure**	**Vapour pressure (VP) of chemical substance / agent / ingredient / component**	*Provide the vapour pressure of the substance at 20 degrees celsius. Note in the data field „Other comments on chemical substance / agent / ingredient / component“ in case the inserted vapour pressure relates to a temperature other than 20 degrees celsius. If you want to indicate that the substance is non-volatile without specifying the vapor pressure further, this is stated by inserting "-1". If you can't assign a vapour pressure, because you can't retrieve it from publication or a sum of different substance was measured, this is stated by inserting "-2".*
field_ag_concentra_product	**Concentration of chemical substance / agent / ingredient / component in chemical product**	*Indicate the concentration of the chemical substance / agent / ingredient / component in the chemical product. The data field accept numerical input only. If the concentration information is only available in text form, e.g. if a concentration range is to be specified (e.g. 0.5 - 5 % or mean concentration: 5 % +/- 2%), then enter the available information on the concentration in the data field "Other comments on chemical substance / agent / ingredient / component".*

field_ag_unit_concentra_product	**Unit concentration of chemical substance / agent / ingredient / component in chemical product**	*Outline the unit in which the concentration of the chemical substance / agent / ingredient / component in the used chemical product is inserted in DermExpoDB, i.e. g/l, g/kg, weight-%, volume-%.*
field_ag_concentra_formulation	**Concentration of chemical substance in chemical formulation / application solution / mixture**	*Indicate the concentration of the chemical substance / agent / ingredient / component in the chemical formulation / application solution / mixture. If the pure chemical product was used during the measurement, this data field can either remain empty or should match the entry in the data field "Concentration of the chemical substance / agent / ingredient / component in chemical product". The data field accept numerical input only. If the concentration information is only available in text form, e.g. if a concentration range is to be specified (e.g. 0.5 - 5 % or mean concentration: 5 % +/- 2%), enter the available information on the concentration in the data field "Other comments on chemical substance / agent / ingredient / component".*
field_ag_unit_conc_formulation	**Unit concentration of chemical substance in chemical formulation / application solution / mixture**	*Outline the unit in which the concentration of the chemical substance / agent / ingredient / component in the used chemical formulation / application solution / mixture is uploaded in DermExpoDB, i.e. g/l, g/kg, weight-%, volume-%.*
field_ag_comments	**Other comments on chemical substance / agent / ingredient / component**	*Address any other information that may be relevant and related to the chemical substance / agent / ingredient / component measured during the measurement.*
**field_ffu_product_category**	**Product category**	*Describe the appearance of the chemical product used in the measurement as it was prior to use or processing. For example, is the product a liquid concentrate, a solid object, a powder, a paste etc. Choose from predefined categories.*
**field_ffu_product_type**	**Product type**	*Describe the appearance of the chemical product used in the measurement as it was prior to use or processing. For example, is the product a liquid concentrate, a solid object, a powder, a paste etc. Choose from predefined categories. It is possible to select more than one element from the predefined list.*

**field_ffu_solution_type**	**Chemical formulation / application solution / mixture type**	*Describe the appearance of the chemical formulation / application solution / mixture during use or processing. For example is the pure product handled or a treated object or a dilution of liquid concentrate etc. Choose from predefined categories. It is possible to select more than one element from the predefined list.*
field_ffu_solution_density	**Density of applied chemical product / formulation / application solution / mixture**	*Specify the density of the applied chemical product / formulation / application solution / mixture in g/L. The data field accept numerical input only. If the density information is only available in text form, e.g. if a density range is to be specified (e.g. 790-900 g/l), enter the available information on the density in the data field "Other comments on the chemical substance / agent / ingredient / component".*
field_ffu_solvent	**Solvent**	*If applicable, specify the name of the (primary) substance(s) / agent(s) / ingredient(s) / component(s) that was / were used as solvent(s) in the product.*
field_ffu_dustiness	**Dustiness**	Only relevant for solid products: Specify the dustiness of the product / material. Predefined categories are available as well as a free text option.
field_ffu_dustiness_other	**Dustiness - Free text**	
field_ffu_dustiness_method	**Dustiness test method**	Only relevant for solid products: Describe the method used to determine the dustiness of a product / material including all relevant details.
field_ffu_moist_content	**Moisture content**	Only relevant for solid products: Indicate the moisture content of the chemical product / material. This does not apply to wetting during the activity. Predefined categories are available as well as a free text option.
field_ffu_moist_content_other	**Moisture content - Free text**	
field_ffu_viscosity	**Viscosity**	*Only relevant for liquid products: Indicate the viscosity of the used chemical product / formulation / application solution / mixture. Predefined categories are available as well as a free text option.*
field_ffu_viscosity_other	**Viscosity - Free text**	

field_ffu_comments	**Other comments on chemical product / formulation / application solution / mixture**	*Address any other information that may be relevant and related to the chemical product / formulation / application solution / mixture used during the measurement.*
**field_spl_study_design**	**Study design**	*Specify the type of study (i.e. field, intervention, laboratory/experimental study) and whether the study was conducted under normal or influenced working conditions. If the study consisted of several different parts, e.g. a laboratory study followed by workplace measurements, then only the type of study relevant to the measured data at hand should be indicated. Choose from predefined categories.*
**field_spl_smpl_mthd_pot_hand**	**Sampling method potential dermal hand exposure**	*Specify the sampling method used to measure the specified exposure. Choose from predefined categories. Familiarize yourself with the definitions for potential and actual exposure as well as body and hand exposure outlined in "A guide to insert and manage content in DermExpoDB".*
**field_spl_smpl_mthd_act_hand**	**Sampling method actual dermal hand exposure**	*Specify the sampling method used to measure the specified exposure. Choose from predefined categories. Familiarize yourself with the definitions for potential and actual exposure as well as body and hand exposure outlined in "A guide to insert and manage content in DermExpoDB".*
**field_spl_smpl_mthd_pot_body**	**Sampling method potential dermal body exposure**	*Specify the sampling method used to measure the specified exposure. Choose from predefined categories. Familiarize yourself with the definitions for potential and actual exposure as well as body and hand exposure outlined in "A guide to insert and manage content in DermExpoDB".*
**field_spl_smpl_mthd_act_body**	**Sampling method actual dermal body exposure**	*Specify the sampling method used to measure the specified exposure. Choose from predefined categories. Familiarize yourself with the definitions for potential and actual exposure as well as body and hand exposure outlined in "A guide to insert and manage content in DermExpoDB".*
field_spl_derm_ql_standards	**Quality standard for dermal sampling**	*If applicable, indicate the quality standards according to which the study was carried out. This can be, for example, a standard or a reference to a publication or a previously performed study. Predefined categories are available as well as a free text option.*
field_spl_dermal_ql_standards_ot	**Quality standard for dermal sampling - Free text**	

field_spl_surf_area_method	**Method to determine the (sampler or body part) surface area**	*Outline the methodology used to determine the surface area of the samplers for the various body parts. If the method is standardized or published in a peer-reviewed publication, a reference to the standard or publication is sufficient. When the details of the methodology are not standardized or the surface area of the samplers or the methodology used to determine them are unique to the study, a detailed description of the method should be provided (e.g. by weighing, reference to standard body areas). For summary statistics data, the surface areas of all relevant samplers (e.g. samplers for the hands and different body parts) should be provided here. For raw data, individual data fields for the different samplers exist and thus do not need to be stated here. If the methods for hand and body exposure are different, both must be entered in this data field.*
field_spl_quant_limit_type	**Type of quantification limit**	*Specify any quantification limit estimated in the study, e.g. limit of detection (LOD), limit of quantification (LOQ), recovery rate or other for the analytical and / or sampling methods.*
field_spl_smpl_mthd_pot_hand_dtl	**Sampling method potential dermal hand exposure - details**	*Provide all relevant details for the method used to sample the specified exposure. This thereby includes all relevant information on transport and storage. Relevant information may be number of samples, size of patches, position of the patches, sampling material used, details on impregnation of patches, suppliers etc. Familiarise yourself with the definitions of body and hand exposure as well as potential and actual exposure presented in "A guide to insert and manage content in DermExpoDB".*
field_spl_smpl_mthd_act_hand_dtl	**Sampling method actual dermal hand exposure - details**	*Provide all relevant details for the method used to sample the specified exposure. This thereby includes all relevant information on transport and storage. Relevant information may be number of samples, size of patches, position of the patches, sampling material used, details on impregnation of patches, suppliers etc. Familiarise yourself with the definitions of body and hand exposure as well as potential and actual exposure presented in "A guide to insert and manage content in DermExpoDB".*
field_spl_ana_methd_hand	**Analytical method dermal hand exposure**	*Provide all relevant details of the analytical method used to determine the specified exposure. This thereby includes all relevant information from the point at which the samples are analyzed in the laboratory or on site. Familiarise yourself with the definitions of body and hand exposure presented in "A guide to insert and manage content in DermExpoDB".*
field_spl_unit_ql_hands	**Unit QL hands**	*Outline the units in which the quantification limits (QL) for the specified sample is inserted in DermExpoDB. Familiarize yourself with the definitions for body and hand exposure outlined in the guide. Predefined categories are available as well as a free text option.*
field_spl_unit_ql_hands_other	**Unit QL hands - Free text**	

field_spl_ql_hands	**Hands QL**	*Provide the quantification limit(s) (QL) for the specified exposure measurement in the unit specified in the data field „Unit QL hands“ or „Unit QL hands - Free text“.*
field_spl_smpl_mthd_pot_body_dtl	**Sampling method potential dermal body exposure - details**	*Provide all relevant details for the method used to sample the specified exposure. This thereby includes all relevant information on transport and storage. Relevant information may be number of samples, size of patches, position of the patches, sampling material used, details on impregnation of patches, suppliers etc. Familiarise yourself with the definitions of body and hand exposure as well as potential and actual exposure presented in "A guide to insert and manage content in DermExpoDB".*
field_spl_smpl_mthd_act_body_dtl	**Sampling method actual dermal body exposure - details**	*Provide all relevant details for the method used to sample the specified exposure. This thereby includes all relevant information on transport and storage. Relevant information may be number of samples, size of patches, position of the patches, sampling material used, details on impregnation of patches, suppliers etc. Familiarise yourself with the definitions of body and hand exposure as well as potential and actual exposure presented in "A guide to insert and manage content in DermExpoDB".*
field_spl_ana_mthd_body	**Analytical method dermal body exposure**	*Provide all relevant details of the analytical method used to determine the specified exposure. This thereby includes all relevant information from the point at which the samples are analyzed in the laboratory or on site. Familiarise yourself with the definitions of body and hand exposure presented in "A guide to insert and manage content in DermExpoDB".*
field_spl_unit_ql_body	**Unit QL body**	*Outline the units in which the quantification limits (QL) for the specified sample is inserted in DermExpoDB. Familiarize yourself with the definitions for body and hand exposure outlined in the guide. Predefined categories are available as well as a free text option.*
field_spl_unit_ql_body_other	**Unit QL body - Free text**	
field_spl_ql_head	**Head / face QL**	*Provide the quantification limit(s) (QL) for the specified exposure measurement in the unit specified in the data field „Unit QL body“ or „Unit QL body - Free text“.*
field_spl_ql_neck	**Neck QL**	*Provide the quantification limit(s) (QL) for the specified exposure measurement in the unit specified in the data field „Unit QL body“ or „Unit QL body - Free text“.*
field_spl_ql_front	**Front QL**	*Provide the quantification limit(s) (QL) for the specified exposure measurement in the unit specified in the data field „Unit QL body“ or „Unit QL body - Free text“.*
field_spl_ql_back	**Back QL**	*Provide the quantification limit(s) (QL) for the specified exposure measurement in the unit specified in the data field „Unit QL body“ or „Unit QL body - Free text“.*
field_spl_ql_arms	**Arms QL**	*Provide the quantification limit(s) (QL) for the specified exposure measurement in the unit specified in the data field „Unit QL body“ or „Unit QL body - Free text“.*

field_spl_ql_upper_arms	**Upper arms QL**	*Provide the quantification limit(s) (QL) for the specified exposure measurement in the unit specified in the data field „Unit QL body" or „Unit QL body - Free text".*
field_spl_ql_forearms	**Forearms QL**	*Provide the quantification limit(s) (QL) for the specified exposure measurement in the unit specified in the data field „Unit QL body" or „Unit QL body - Free text".*
field_spl_ql_legs	**Legs QL**	*Provide the quantification limit(s) (QL) for the specified exposure measurement in the unit specified in the data field „Unit QL body" or „Unit QL body - Free text".*
field_spl_ql_thighs	**Thighs QL**	*Provide the quantification limit(s) (QL) for the specified exposure measurement in the unit specified in the data field „Unit QL body" or „Unit QL body - Free text".*
field_spl_ql_lower_legs	**Lower legs QL**	*Provide the quantification limit(s) (QL) for the specified exposure measurement in the unit specified in the data field „Unit QL body" or „Unit QL body - Free text".*
field_spl_ql_feet	**Feet QL**	*Provide the quantification limit(s) (QL) for the specified exposure measurement in the unit specified in the data field „Unit QL body" or „Unit QL body - Free text".*
field_spl_smpl_mthd_inhal	**Sampling method inhalation exposure**	*Specify whether personal or stationary sampling was performed. Choose from predefined categories.*
field_spl_smpl_mthd_inhal_dtl	**Sampling method inhalation exposure - details**	*Provide all relevant details for the chosen inhalation sampling method. This thereby includes all relevant information on transport and storage. Other relevant information may be type of sampling material, type of sampling head, flow rate etc.*
field_spl_ana_methd_inhal	**Analytical method inhalation exposure**	*Provide all relevant details of the analytical method used to determine the specified exposure. This thereby includes all relevant information from the point at which the samples are analyzed in the laboratory or on site.*
field_spl_unit_ql_inhal	**Unit QL inhalation exposure**	*Outline the units in which the quantification limits (QL) for the specified sample is inserted in DermExpoDB. Predefined categories are available as well as a free text option.*
field_spl_unit_ql_inhal_other	**Unit QL inhalation exposure - Free text**	
field_spl_inhal_ql	**Inhalation exposure QL**	*Provide the quantification limit(s) (QL) for the specified exposure measurement in the unit specified in the data field „Unit QL body" or „Unit QL body - Free text".*
field_spl_smpl_mthd_surface	**Sampling method surface contamination**	*Provide all relevant details for the chosen surface contamination sampling method. Relevant information may be number of samples, location of samples, surface area of samples, sampling material, pressure applied, number of wipes per sample etc. This thereby includes all relevant information on transport and storage. Enter any relevant quantification limit (QL) (including the unit) in this data field.*

field_spl_ana_mthd_surface	**Analytical method surface contamination**	*Provide all relevant details of the analytical method used to determine the specified exposure. This thereby includes all relevant information from the point at which the samples are analyzed in the laboratory or on site.*
field_spl_smpl_mthd_oral	**Sampling method oral exposure**	*Provide all relevant details for the chosen oral sampling method. This thereby includes all relevant information on transport and storage. Enter any relevant quantification limit (QL) in this data field.*
field_spl_ana_mthd_oral	**Analytical method oral exposure**	*Provide all relevant details of the analytical method used to determine the specified exposure. This thereby includes all relevant information from the point at which the samples are analyzed in the laboratory or on site.*
field_spl_smpl_mthd_biomon	**Sampling method biomonitoring**	*Provide all relevant details for the chosen biomonitoring sampling method. This thereby includes all relevant information on transport and storage. Enter any relevant quantification limit (QL) (including the unit) in this data field.*
field_spl_ana_mthd_biomon	**Analytical method biomonitoring**	*Provide all relevant details of the analytical method used to determine the specified exposure. This thereby includes all relevant information from the point at which the samples are analyzed in the laboratory or on site.*
field_spl_comments	**Other comments on sampling**	*Insert any additional information with regard to sampling and analytics and statistical analysis of data.*
**field_data_owner**	**Dataset owner**	*Specify the institute that owns the data or the institute that has been responsible for extracting published data into the DermExpoDB format.*
**field_data_entry**	**Date of data entry**	*Specify the date on which the data was inserted into DermExpoDB.*
**field_sampling_strategy**	**Sampling strategy**	*Indicate whether the sampling was carried out for a specific activity / task / process, shift-based, according to an experimental protocol or according to any other predefined period. In case of pre-shift data, choose „pre-shift“. Choose from predefined categories.*
**field_exposure_route**	**Exposure route(s)**	*Indicate whether the measured data is dermal, inhalation or biomonitoring data. For dermal data, distinguish whether data related to hand or body exposure. Familiarize yourself with the definitions for body and hand exposure outlined in "A guide to insert and manage content in DermExpoDB". Choose from predefined categories. It is possible to select more than one element from the predefined list.*
field_sampling_period	**Sampling period**	*Give details of the sampling period, i.e. in which year or month or day or over which period (2-week period) the sampling took place.*
field_derm_sampling_moment	**Dermal sampling moment**	*Indicate the moment during the (working) day when the sample was collected (e.g. beginning of shift, mid shift, end of shift). Choose from predefined categories.*

field_sampling_time	**Sampling time**	*Give details with regard to the duration of sampling, i.e. mean, median and/or range of sampling time (preferably in min).*
field_company_nbr	**Company / sampling site number**	*Indicate the number of companies / sampling sites and/or the unique codes for the investigated companies / sampling sites involved in the measurement preferably as indicated in the reference.*
field_person_nbr	**Person number**	*Indicate the number of workers / volunteers / persons and/or the unique codes for the workers / volunteers / persons involved in the measurement preferably as indicated in the reference.*
field_applied_ subst_amount_sd	**Applied amount of substance / agent / ingredient / component**	*Specify the average, median and/or range of amount / quantity /volume of the chemical substance / agent / ingredient / component (preferably in kg für solids or l for liquids) that was applied during the measurement. Please also include the relevant unit.*
field_applied_solution_amount_sd	**Applied amount of chemical formulation / application solution / mixture**	*Specify the average, median and/or range of amount / quantity /volume of the chemical formulation / application solution / mixture (preferably in kg für solids or l for liquids) that was applied during the measurement. Please also include the relevant unit.*
field_body_part	**Sampled body part**	*Specify the sampled body part. Familiarize yourself with the definitions for hand and body exposure as outlined in "A guide to insert and manage content in DermExpoDB".*
field_potential_actual_exposure	**Potential / actual exposure**	*Indicate whether potential, actual, potential and actual or covered skin exposure was measured. If type of exposure is not known, insert „not known“. For example, this often applies to neck and wrist measurements, where clothing may have covered part of the samplers. If inhalation exposure or pre-shift exposures were measured, insert „not relevant“. Familiarize yourself with the definitions for potential and actual exposure as outlined in "A guide to insert and manage content in DermExpoDB". Choose from predefined categories.*
field_number_of_samples	**Number of samples**	*Enter the number of samples on the basis of which the statistical values were calculated.*
field_sampling_unit	**Unit of statistical parameters**	*Outline the unit in which the statistical parameters for the collected samples are inserted in DermExpoDB. Predefined categories are available as well as a free text option.*
field_sampling_unit_other	**Unit of statistical parameters - Free text**	

field_am	**AM**	*Provide the value of the specified statistical parameter in the unit specified in the data field "Unit of statistical parameters". Unless otherwise stated in the data field „Unit of statistical parameters", the given exposure relates to the chemical substance / agent / ingredient / component and body part of interest. If the value of the specified statistical parameter is below the quantification limit (QL), this is stated by inserting "-1". Specify the QL value in the QL data field for the corresponding body part.*
field_sd	**SD**	*Provide the value of the specified statistical parameter in the unit specified in the data field "Unit of statistical parameters". Unless otherwise stated in the data field „Unit of statistical parameters", the given exposure relates to the chemical substance / agent / ingredient / component and body part of interest. If the value of the specified statistical parameter is below the quantification limit (QL), this is stated by inserting "-1". Specify the QL value in the QL data field for the corresponding body part.*
field_median	**Median**	*Provide the value of the specified statistical parameter in the unit specified in the data field "Unit of statistical parameters". Unless otherwise stated in the data field „Unit of statistical parameters", the given exposure relates to the chemical substance / agent / ingredient / component and body part of interest. If the value of the specified statistical parameter is below the quantification limit (QL), this is stated by inserting "-1". Specify the QL value in the QL data field for the corresponding body part.*
field_gm	**GM**	*Provide the value of the specified statistical parameter in the unit specified in the data field "Unit of statistical parameters". Unless otherwise stated in the data field „Unit of statistical parameters", the given exposure relates to the chemical substance / agent / ingredient / component and body part of interest. If the value of the specified statistical parameter is below the quantification limit (QL), this is stated by inserting "-1". Specify the QL value in the QL data field for the corresponding body part.*
field_gsd	**GSD**	*Provide the value of the specified statistical parameter in the unit specified in the data field "Unit of statistical parameters". Unless otherwise stated in the data field „Unit of statistical parameters", the given exposure relates to the chemical substance / agent / ingredient / component and body part of interest. If the value of the specified statistical parameter is below the quantification limit (QL), this is stated by inserting "-1". Specify the QL value in the QL data field for the corresponding body part.*
field_minimum	**Minimum**	*Provide the value of the specified statistical parameter in the unit specified in the data field "Unit of statistical parameters". Unless otherwise stated in the data field „Unit of statistical parameters", the given exposure relates to the chemical substance / agent / ingredient / component and body part of interest. If the value of the specified statistical parameter is below*

		the quantification limit (QL), this is stated by inserting "-1". Specify the QL value in the QL data field for the corresponding body part.
field_maximum	**Maximum**	*Provide the value of the specified statistical parameter in the unit specified in the data field "Unit of statistical parameters". Unless otherwise stated in the data field „Unit of statistical parameters“, the given exposure relates to the chemical substance / agent / ingredient / component and body part of interest. If the value of the specified statistical parameter is below the quantification limit (QL), this is stated by inserting "-1". Specify the QL value in the QL data field for the corresponding body part.*
field_5th_percentile	**5th percentile**	*Provide the value of the specified statistical parameter in the unit specified in the data field "Unit of statistical parameters". Unless otherwise stated in the data field „Unit of statistical parameters“, the given exposure relates to the chemical substance / agent / ingredient / component and body part of interest. If the value of the specified statistical parameter is below the quantification limit (QL), this is stated by inserting "-1". Specify the QL value in the QL data field for the corresponding body part.*
field_10th_percentile	**10th percentile**	*Provide the value of the specified statistical parameter in the unit specified in the data field "Unit of statistical parameters". Unless otherwise stated in the data field „Unit of statistical parameters“, the given exposure relates to the chemical substance / agent / ingredient / component and body part of interest. If the value of the specified statistical parameter is below the quantification limit (QL), this is stated by inserting "-1". Specify the QL value in the QL data field for the corresponding body part.*
field_25th_percentile	**25th percentile**	*Provide the value of the specified statistical parameter in the unit specified in the data field "Unit of statistical parameters". Unless otherwise stated in the data field „Unit of statistical parameters“, the given exposure relates to the chemical substance / agent / ingredient / component and body part of interest. If the value of the specified statistical parameter is below the quantification limit (QL), this is stated by inserting "-1". Specify the QL value in the QL data field for the corresponding body part.*
field_75th_percentile	**75th percentile**	*Provide the value of the specified statistical parameter in the unit specified in the data field "Unit of statistical parameters". Unless otherwise stated in the data field „Unit of statistical parameters“, the given exposure relates to the chemical substance / agent / ingredient / component and body part of interest. If the value of the specified statistical parameter is below the quantification limit (QL), this is stated by inserting "-1". Specify the QL value in the QL data field for the corresponding body part.*

field_90th_percentile	**90th percentile**	*Provide the value of the specified statistical parameter in the unit specified in the data field "Unit of statistical parameters". Unless otherwise stated in the data field „Unit of statistical parameters", the given exposure relates to the chemical substance / agent / ingredient / component and body part of interest. If the value of the specified statistical parameter is below the quantification limit (QL), this is stated by inserting "-1". Specify the QL value in the QL data field for the corresponding body part.*
field_95th_percentile	**95th percentile**	*Provide the value of the specified statistical parameter in the unit specified in the data field "Unit of statistical parameters". Unless otherwise stated in the data field „Unit of statistical parameters", the given exposure relates to the chemical substance / agent / ingredient / component and body part of interest. If the value of the specified statistical parameter is below the quantification limit (QL), this is stated by inserting "-1". Specify the QL value in the QL data field for the corresponding body part.*
field_samples_below_ql	**% of samples below QL**	*Report the percentage (%) of samples with values below the limit of quantification (QL) that were included in the calculation of the statistical parameters of the measured data.*
field_summary_comments	**Other comments on summary statistics data**	*Address any other information that may be relevant and related to the measured data, e.g. when measured values were adjusted in some way.*

Mandatory fields are marked in bold in the 'Machine Name' column.

Supplementary Material 7 – A guide to insert and manage content in DermExpoDB

The guide is intended to help import dermal data into the DermExpoDB software and to manage the data in the software.

Paragrahs:
The content to be inserted in DermExpoDB is structured into the following paragaphs:

- Reference
- Activity
- Workplace
- Control measures
- Chemical formulation / application solution / mixture
- Chemical substance / agent / ingredient / component
- Sampling
- Data

Data field types:
There are different data types for different data fields:

- Data fields for free text input (free text)
- Data fields for only numeric input (unit defined in the header)
- Data fields for date entry only
- Categorical data fields:
    - Categorical data fields with a predefined choice only (not editable for user, but for admins) (categorical)
    - Categorical data fields with one option being free text. Selecting this category will open another free text data field (two stage data fields)
    - Categorical field with multi-selection possiblity and additional free text field (multi-field)

Data types accepted in DermExpoDB:

Two different types of data can be uploaded in DermExpoDB:

1. Raw data: DermExpoDB accepts the upload of single / individual measurement data. This is the exposure of a specific worker / volunteer / person at a specific point in time and place. Prior to the upload it must be ensured that the data has been anonymized i.e. no conclusions can be drawn about the sampled workers / volunteers / persons and the company / sampling site.
2. Summary statistics data: DermExpoDB also accepts the upload of statistical values calculated on the basis of a data collective. The data collective usually comprises measured exposures for a specific exposure situation but for several workers / volunteers / persons and/or companies / sampling sites and/or points in time.

Data import:
The data can be imported into the software in two ways. In the content section of the software the

selection "Add content" leads to two forms that can be filled in manually. The selection of the form type depends on whether raw data or summary statistics data (e.g. from literature) are to be imported. In addition there is the possibility to import the data via a csv file which can be uploaded into the system via the "Importer" section. Data upload fails in case mandatory information are missing. Warning messages are issued for information that is missing but be regarded as relevant information by the DermExpoDB developers. Warnings do not lead to a rejection of the upload.

Mandatory data entries:
The data entries marked with a red asterisk are mandatory. These fields must be filled in otherwise the record cannot be saved. The reason for the mandatory fields is that these fields are used for DermExpoDB's search filters or are needed for a public search for further information of the dataset. The name of each record is assigned via the data field "Experiment name" (raw data) / “Dataset name” (summary statistics data). The data field is important to find and access data in the software. A meaningful unique and unambiguous naming of each data set is therefore recommended.

Confidential / non-confidental data:
Data owners can highlight their data as confidential data. Outside Parties may request access to protected but non-confidential data only. For those parties having full access to DermExpoDB the use (even of small amounts) of confidential data without approval is not permitted.

Handling of missing information:
If certain data fields cannot be populated e.g. because no related information is available or the type of information does not fit the case at hand (e.g. specification of a spraying pressure for a refilling activity) then the corresponding fields should be left blank. Do not enter NA or NR or similar. For the mandatory fields there is always an option "other" or "not known" available. Mandatory free text fields must be populated with text.

Reference to other publications in DermExpoDB:
The original reference in which the measurement data are presented may contain references to other publications (hereafter referred to as secondary references) that may need to be referenced in a data field of DermExpoDB. It seems to be sufficient that these secondary references are found with the help of the original reference so that a reference in DermExpoDB such as Fritz et al. 2005 seems to be sufficient. If the secondary reference is not cited in the original reference it must be ensured that the secondary reference is traceable by the users of DermExpoDB.

Special characters:
Large amounts of data can be imported into the system via csv files. The different data entries in these csv files may be separated by commas or semicolons. However these special characters should then be avoided as part of a data entry to avoid rejection of the upload. Categorical fields in the software application are necessary to allow a quick search for users e.g. via the search function and the predefined filters therein. For information that cannot be entered in the appropriate data field due to the nature of the data field type there is an additional comment field for each paragraph. We recommend not to write numbers in the scientific notation and to express superscript numbers as follows: m2 as m2 or m-2 as m-2.

Log-Function:
Some information is automatically collected and stored by the application during data upload:

- Date and time of data upload
- Institute responsible for data upload

- Dates and times of amendment
- Data type (raw data vs summary statistics data)

Filter fields in DermExpoDB:
In the "Search" section of the software it is possible to search for data with certain properties. For this purpose the following data fields have been identified which can be searched for specific content:

- Data owner
- Reference title
- Author(s)
- Year of publication
- Study quality
- Study design
- Sampling strategy
- Sampling method potential hand exposure
- Sampling method actual hand exposure
- Sampling method potential body exposure
- Sampling method actual body exposure
- Activity
- Activity description
- PROC Number & PROC description
- Location
- Product category
- Product type
- Chemical formulation /application solution / mixture type
- CAS
- Vapour pressure

All of these data fields are mandatory and often categorical. In order to give the user the opportunity to enter all relevant information in DermExpoDB there is either the option of inserting additional free text in the respective data field in separate comment fields. In addition two other filters are generated automatically by the system based on the information available for the data sets. Firstly it is possible to filter by data type (raw data vs summary statistics data). Second the system automatically recognizes if inhalation exposure values body exposure values hand exposure values or biomonitoring exposure values are available for a data set. This is done checking whether the corresponding data fields are empty or not. Therefore it is immensely important that in case of missing measured values these data fields are not filled with NA or NR or similar placeholder expressions.

User support:
Short explanations are available for each data field. These can be found in the software via the "i" next to the corresponding data fields or in the appendix to this document. It is possible to contact the administrators of the software application if support is needed or to report errors in the software or data.

Supplementary Material 8 – Definition of terms in DermExpoDB

Potential exposure	Potential exposure refers to exposure on the protective clothing or on the skin when no protective clothing is worn.
Actual exposure	Actual exposure refers to exposure underneath protective clothing.
Covered skin	Covered skin exposure refers to exposure unterneath clothing which is no protective clothing.
Body exposure	Body exposure refers to exposures to the face/head neck trunk arms legs and feet but not to the hands. In DermExpoDB the wrists are considered part of the forearms and are thus included as body exposure and not as hand exposure.
Hand exposure	Hand exposure refers to exposure to the hands whereby exposure of the entire hands or a part of the hands e.g. palms ore forefingers can be meant.

Note: When not stated otherwise the given exposure is related to the agent and body part of interest.

Supplementary Material 9 – Abbreviations used in DermExpoDB

ACH	Air changes per hour
ADE	Actual dermal exposure
AM	Statistical parameter: arithmetic mean of measured exposure in a data collective
CAS	Chemical Abstracts Service Number
DOI	Digital object identifier
GM	Statistical parameter: geometric mean / median of measured exposure in a data collective
GSD	Statistical parameter: geometric stand-ard deviation of measured exposure in a data collective
ISBN	International Standard Book Number
LOD	Limit of detection
LOQ	Limit of quantification
Max	Statistical parameter: maximum of measured exposure in a data collective
Min	Statistical parameter: minimum of measured exposure in a data collective
PDE	Potential dermal exposure
PROC	Process categories as used under REACH
QL	Quantification limit (in general terms)
SA	Sample area
SD	Statistical parameter: standard deviation of measured exposure in a data collective
VP	Vapour pressure

Supplementary Material 10 – FAIR self-assessment of DermExpoDB

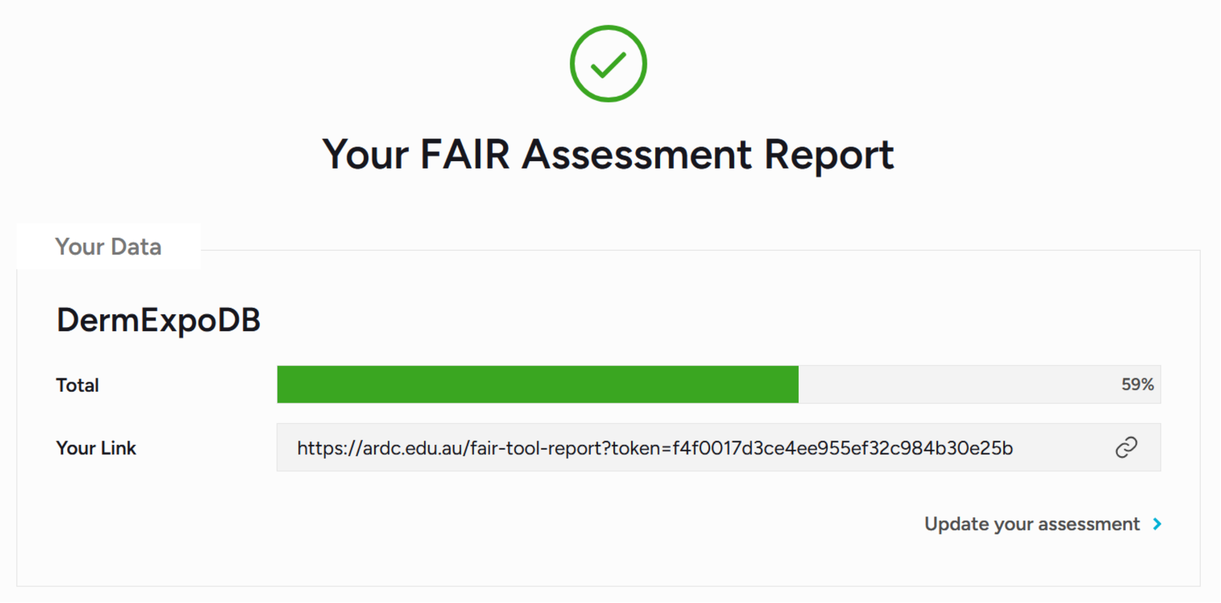

Link: https://ardc.edu.au/fair-tool-report?token=f4f0017d3ce4ee955ef32c984b30e25b
